# Skull and partial skeleton of a new pachycetine genus (Cetacea, Basilosauridae) from the Aridal Formation, Bartonian middle Eocene, of southwestern Morocco

**DOI:** 10.1371/journal.pone.0276110

**Published:** 2022-10-26

**Authors:** Philip D. Gingerich, Ayoub Amane, Samir Zouhri

**Affiliations:** 1 Museum of Paleontology, University of Michigan, Ann Arbor, Michigan, United States of America; 2 Department of Geology and Health and Environment Laboratory, Faculty of Sciences Aïn Chock, Hassan II University of Casablanca, Casablanca, Morocco; Royal Belgian Institute of Natural Sciences, BELGIUM

## Abstract

*Pachycetus paulsonii*, *Pachycetus wardii*, and *Antaecetus aithai* are middle Eocene archaeocete whales found in Europe, North America, and Africa, respectively. The three are placed in the new basilosaurid subfamily Pachycetinae. *Antaecetus* is a new genus known from Egypt and Morocco, and the only pachycetine known from a substantial postcranial skeleton. The skull of *A*. *aithai* described here resembles that of *Saghacetus osiris* in size, but lacks the narrowly constricted rostrum of *Saghacetus*. *Antaecetus* is smaller than *Pachycetus* and its teeth are more gracile. Upper premolars differ in having two rather than three accessory cusps flanking the principal cusp. Pachycetines differ from dorudontines in having elongated posterior thoracic and lumbar vertebrae like those of *Basilosaurus*, but differ from basilosaurines and from dorudontines in having conspicuously pachyosteosclerotic vertebrae with dense and thickly laminated cortical bone surrounding a cancellous core. Pachycetinae are also distinctive in having transverse processes on lumbar vertebrae nearly as long anteroposteriorly as the corresponding centrum. We infer from their pachyosteosclerotic vertebrae that pachycetines were probably sirenian-like slow swimmers living in shallow coastal seas and feeding on passing fish and mobile invertebrates.

## Introduction

The cetacean family Basilosauridae is a cosmopolitan, fully-aquatic group of archaic whales or archaeocetes ranging in age from the late middle Eocene (latest Lutetian or early Bartonian stage-age) through the late Eocene (Priabonian stage-age). Most basilosaurids come from northern hemisphere localities in North Africa, Asia, Europe, and North America [1–4], but basilosaurids are also known from South America and Antarctica in the southern hemisphere [5, 6]. The temporal range of basilosaurids spans an interval from about 41 to 34 million years before present [7].

Fifteen genera and twenty-three species of Basilosauridae appear to be valid (Table 1). *Basilosaurus cetoides*, *Zygorhiza kochii*, *Dorudon atrox*, and *Pontogeneus peruvianus* are the four genera and species for which good skulls and associated skeletons have been described [1, 8, 9], and much of our understanding of Basilosauridae is based on these specimens. *Pontogeneus* is the appropriate generic name for *P*. *brachyspondylus* [1], and by extension *P*. *peruvianus* [10].

**Table 1:** Temporal and geographic distribution of three subfamilies, 15 genera, and 23 species of Eocene Basilosauridae.

Genus and species	Species author, year, and page	Holotype	Age	Type locality	North latitude	East longitude
**Basilosaurinae**						
*Basilosaurus cetoides*	Owen, 1841: 69 [11]	ANSP 12944A	Priabonian	Ouachita River, Louisiana, U.S.A.	31.92800	−91.94000
*Basilosaurus isis*	Beadnell in Andrews, 1904: 214 [12]	CGM 10208	Priabonian	Birket Qarun, Fayum, Egypt	29.47200	30.35900
*Eocetus schweinfurthi*	Fraas, 1904: 217 [13]	SMNS 10986	Bartonian	Gebel Mokattam, Cairo, Egypt	30.02200	31.27300
*Eocetus drazindai*	Gingerich et al., 1997: 57 [14]	GSP-UM 3193	Bartonian	Bari Nadi, Punjab, Pakistan	30.78283	70.42783
*Basiloterus hussaini*	Gingerich et al., 1997: 62 [14]	GSP-UM 3190	Bartonian	Bari Nadi, Punjab, Pakistan	30.78783	70.44050
**Dorudontinae**						
*Dorudon serratus*	Gibbes, 1845: 254 [15]	MCZ 8763	Priabonian	Santee Canal, S. Carolina, U.S.A.	33.26500	−79.96000
*Zygorhiza kochii*	Reichenbach, 1847: 13 [16]	MNB Ma-43248	Priabonian	Uncertain, Alabama, U.S.A.	31.68960	−88.28170
*Pontogeneus brachyspondylus*	Müller, 1849: 26 [17]	MNB unknown	Priabonian	Uncertain, Alabama, U.S.A.	31.68960	−88.28170
*Saghacetus osiris*	Dames, 1894: 204 [18]	MNB 28388	Priabonian	Garet el-Esh, Fayum, Egypt	29.57100	30.56500
*Dorudon atrox*	Andrews, 1906: 255 [19]	CGM 9319	Priabonian	12 km WSW Garet Gehannam, Egypt	29.27300	30.03100
*Ancalacetus simonsi*	Gingerich and Uhen, 1996: 363 [20]	CGM 42290	Priabonian	Wadi Al Hitan WH-81, Fayum, Egypt	29.27374	30.02344
*Chrysocetus healyorum*	Uhen and Gingerich, 2001: 3 [21]	SCSM 87.195	Priabonian	Santee Quarry, Holly Hill, S. Carolina, U.S.	33.27800	-80.42300
*Stromerius nidensis*	Gingerich, 2007: 366 [22]	UM 100140	Priabonian	Garet el-Esh, Fayum, Egypt	29.57195	30.56637
*Masracetus markgrafi*	Gingerich, 2007: 375 [22]	SMNS 11414	Priabonian	Dimeh, Fayum, Egypt	29.53600	30.66900
*Pontogeneus peruvianus*	Martínez and Muizon, 2011: 518 [5]	MNHN.F.PRU 10	Priabonian	Paracas Bay, Ica, Peru	−13.88175	−76.23706
*Supayacetus muizoni*	Uhen et al., 2011: 960 [23]	MUSM 1465	Bartonian	AV-17, Ica, Peru	−14.66678	−75.63515
*Ocucajea picklingi*	Uhen et al., 2011: 963 [23]	MUSM 1442	Bartonian	AV-19, Ica, Peru	−14.66830	−75.63505
*Chrysocetus fouadassii*	Gingerich and Zouhri, 2015: 278 [24]	FASC Bouj-1	Bartonian	Sabkha de Gueran, Boujdour, Morocco	25.12000	−13.89000
**Pachycetinae**						
*Pachycetus paulsonii*	Brandt, 1873: 336 [25]	Lost	Bartonian	Chyhyryn, Cherkasy, Ukraine	49.07300	32.66200
*Pachycetus wardii*	Uhen, 1999: 514 [26]	USNM 310633	Bartonian	Lanier Quarry, Maple Hill, N. Carolina, U.S.	34.62500	−77.67500
*Antaecetus aithai*	Gingerich and Zouhri, 2015: 280 [24]	FASC Bouj-6	Bartonian	Sabkha de Gueran, Boujdour, Morocco	25.07667	−13.90763
**Subfamily incertae sedis**						
*’Zeuglodon’ wanklyni*	Seeley, 1876: 428 [27]	Lost	Bartonian	Barton Cliff, England, U.K.	50.74280	−1.65520
*’Pachycetus’ humilis*	Van Beneden, 1883: 33 [28]	MMGD NsT-94	Bartonian	Helmstedt, Niedersachsen, Germany	52.22900	11.01000

Basilosaurids are sometimes grouped in a single family without division [3, 5, 9, 24], but there is merit, phenetically at least, in subdividing this based on relative elongation of the posterior thoracic, lumbar, and caudal vertebrae. Basilosaurids with long trunk vertebrae (e.g., *Basilosaurus*, *Eocetus*, *Basiloterus*) are placed in Basilosaurinae, and basilosaurids with short trunk vertebrae (e.g., *Dorudon*, *Zygorhiza*, *Pontogeneus*, *Saghacetus*, etc.) are placed in Dorudontinae [8, 23, 29–31].

Here, we review the taxonomic history of the enigmatic basilosaurid genus *Pachycetus* Van Beneden, 1883 [28] and its several nominal species. We recognize that one of these, *P*. *aithai* from the late middle Eocene (Bartonian) of Morocco, represents a new genus *Antaecetus*, which we diagnose with the aid of a new specimen that includes a skull and much of the axial skeleton. Comparison shows that *Pachycetus* and *Antaecetus* together represent a new subfamily, Pachycetinae, of divergently specialized archaeocetes that swam and lived much differently from other basilosaurids.

### Abbreviations

ANSP: Academy of Natural Sciences, Philadelphia, Pennsylvania, U.S.A.
CGM: Egyptian Geological Museum, Cairo, Egypt
CMM: Calvert Marine Museum, Solomons, Maryland, U.S.A.
CMN: Cossack Museum, Novocherkassk, Rostov, Russia
FSAC: Faculté des Sciences Ain Chock, Université Hassan-II de Casablanca, Morocco
GSP-UM: Geological Survey of Pakistan–University of Michigan collection, Quetta, Pakistan
HMS: Heimatmuseum, Schöningen, Niedersachsen, Germany
KOM: Kirovograd Oblast Museum, Ukraine
KRMHA: Kaliningrad Regional Museum of History and Art, Kaliningrad, Russia
MCZ: Museum of Comparative Zoology, Harvard University, Cambridge, Massachusetts, U.S.A.
MMGD: Museum für Mineralogie und Geologie, Dresden, Sachsen, Germany
MNB: Museum für Naturkunde, Berlin, Germany
MNHN: Muséum Nationale d’Histoire Naturelle, Paris, Île-de-France, France
MUSM: Museo de Historia Natural, Universidad Nacional Mayor de San Marcos, Lima, Peru
NCSM: North Carolina Museum of Natural Sciences, Raleigh, North Carolina, U.S.A.
NHML: Natural History Museum, London, England, U.K.
NMNH-P: National Museum of Natural History, Paleontology, Kyiv, Ukraine
NMR: Natuurhistorisch Museum, Rotterdam, Netherlands
SCSM: South Carolina State Museum, Columbia, South Carolina, U.S.A.
SMNS: Staatliches Museum für Naturkunde, Stuttgart, Baden-Württemberg, Germany
TSNU-GM: Geological Museum, Taras Shevchenko National University, Kyiv, Ukraine
UM: University of Michigan Museum of Paleontology, Ann Arbor, Michigan, U.S.A.
USNM: National Museum of Natural History, Washington, D.C., U.S.A.

## Methods

### Nomenclatural acts

The electronic edition of this article conforms to the requirements of the amended International Code of Zoological Nomenclature, and hence the new names contained herein are available under that Code from the electronic edition of this article. This published work and the nomenclatural acts it contains have been registered in ZooBank, the online registration system for the ICZN. The ZooBank LSIDs (Life Science Identifiers) can be resolved and the associated information viewed through any standard web browser by appending the LSID to the prefix "http://zoobank.org/". The LSID for this publication is: urn:lsid:zoobank.org:pub:23753D98-8394-4D59-A327-93A21BB5EEC5. The electronic edition of this work was published in a journal with an ISSN, has been archived, and is available from the following digital repositories: PubMed Central and LOCKSS.

### Permits

No permits were requird for the described study, which complied with all relevant regulations.

## History of study

**Fig 1:**
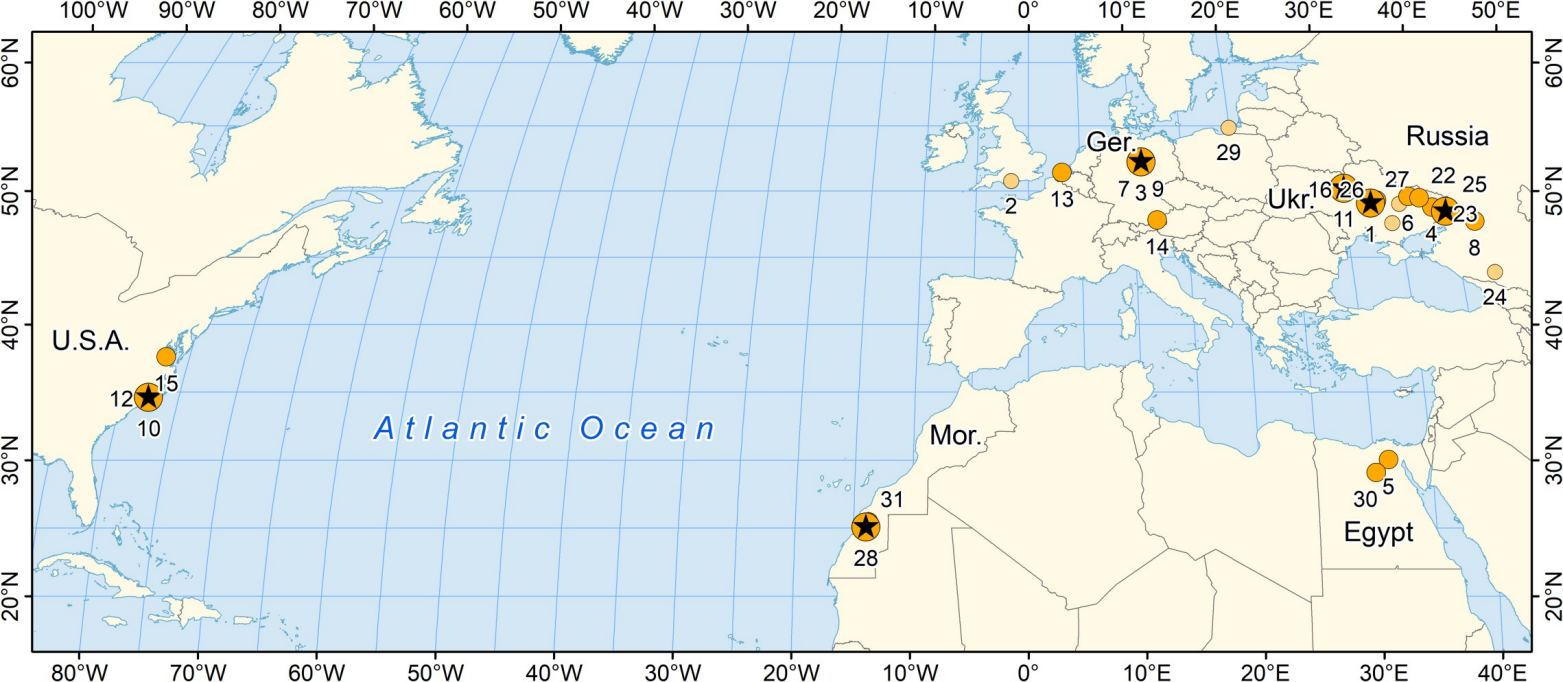
Distribution of Eocene localities yielding *Pachycetus*, *Antaecetus*, or an archaeocete compared to them in Europe, North Africa, and North America. Localities with stars are those yielding type specimens of named species. Larger colored circles represent specimens identified as *Pachycetus*, *Antaecetus*, or a junior synonym. Smaller colored circles are additional records that may represent *Pachycetus* but remain ambiguous. Literature references and coordinates for each locality are listed in Table 2. Base map from Natural Earth (http://www.naturalearthdata.com/).

The history of *Pachycetus* and its constituent and related species is complicated because specimens are relatively rare. Many specimens, including the type specimen of the type species of *Pachycetus*, are isolated vertebrae or small collections of vertebrae. The specimens have been found on three continents. In addition, *Pachycetus* itself was omitted from the most thorough reviews following its publication [1, 32]. Localities yielding *Pachycetus* and its relatives are shown on the map in Fig 1. *Pachycetus*, constituent and related species, and corresponding locality coordinates are listed as they were published in Table 2.

**Table 2:** History of study of Eocene archaeocetes from localities yielding *Pachycetus*, a synonym, or a closely related contemporary.

Author	Year	Page	Identification	Country	Locality	North latitude	East longitude	Map symb.	Map label
Brandt	1873 [25]	336	*Zeuglodon paulsonii*	Ukraine	Chyhyryn	49.07300	32.66200	T	1
Paulson (in Brandt)	1873 [25]	339	Zeuglodon rossicus	Ukraine	Chyhyryn	49.07300	32.66200	—	—
Seeley	1876 [27]	428	*Zeuglodon wanklyni*	England	Barton Cliff	50.74300	−1.65500	O	2
Van Beneden	1883 [28]	32	*Pachycetus robustus*	Germany	Helmstedt	52.22900	11.01000	T	3
Van Beneden	1883 [28]	32	*Pachycetus humilis*	Germany	Helmstedt	52.22900	11.01000	T	—
Lutugin	1894 [33]	147	*Zeuglodon* sp.	Ukraine	Pereshchepnoy	48.76400	38.43900	P	4
Andrews	1907 [34]	124	*Zeuglodon wanklyni*	England	Barton Cliff	50.74300	−1.65500	—	—
Stromer	1908 [35]	109	*Eocetus* schweinfurthi (pt.)	Egypt	Gebel Mokattam	30.02700	31.27300	P	5
Fedorovsky	1912 [36]	280	*Zeuglodon rossicus*	Ukraine	Koropove	49.58900	36.34600	P	6
Kuhn	1935 [37]	223	*’Zeuglodon’* cf. *Z*. *isis*	Germany	Trendelbusch	52.18700	10.97900	P	7
Bogachev	1959 [38]	42	*Zeuglodon paulsonii*	Russia	Khoroshevskaya	47.71900	42.22600	P	8
Halstead and Middleton	1972 [39]	186	*Zygorhiza wanklyni*	England	Barton Cliff	50.74300	−1.65500	—	—
Lienau	1984 [40]	73	*Pachycetus robustus*	Germany	Treue	52.17600	10.98400	P	9
Uhen	1999 [26]	514	*Eocetus wardii*	U.S.A.	Laniers Pit	34.62500	−77.67500	T	10
Gritsenko	2001 [41]	18	*Platyosphys einori*	Ukraine	Pyrohiv, Kyiv	50.21000	30.32000	T	11
Uhen	2001 [42]	3	*Eocetus wardii*	U.S.A.	Rocky Point Quarry	34.42500	−77.86830	P	12
Post	2007 [43]	31	Archaeoceti indet.	Netherlands	Scheur 10	51.41310	3.25150	P	13
Uhen and Berndt	2008 [44]	57	*Eocetus* sp.	Germany	Rohrdorf	47.79694	12.17000	P	14
Weems et al.	2011 [45]	273	*’Eocetus’ wardii*	U.S.A.	Putneys Mill	37.60400	−77.09200	P	15
Gol’din et al.	2012 [46]	105	’*Eocetus’* sp.	Ukraine	Kurenevka, Kyiv	50.49500	30.43100	P	16
Kalmykov	2012 [47]	180	*Basilosaurus* sp.	Russia	Khoroshevskaya	47.71900	42.22600	—	—
Tesakov et al.	2012 [48]	141	*Eocetus* sp.	Russia	Khoroshevskaya	47.71900	42.22600	—	—
Zvonok	2012 [49]	87	Basilosauridae indet.	Ukraine	Nagornoye	49.08300	33.13300	P	17
Zvonok	2012 [49]	88	*Basilosaurus* sp.	Ukraine	Subotiv	49.09200	32.54500	O	18
Zvonok	2012 [49]	88	*Basilosaurus* sp.	Ukraine	Pywycha	49.20200	33.12200	O	19
Zvonok	2012 [49]	88	*Basilosaurus* sp.	Ukraine	Nikopol	47.56900	34.39400	O	20
Zvonok	2012 [49]	88	*Basilosaurus* sp.	Ukraine	Pereshchepyne	49.01500	35.36400	O	21
Zvonok	2012 [49]	88	*Platyosphys paulsoni*	Ukraine	Buhaivka	49.47700	37.38500	P	22
Zvonok	2012 [49]	88	Archaeoceti	Ukraine	Luhansk	48.50600	39.39300	O	23
Zvonok	2012 [49]	88	Basilosauridae indet.	Russia	Pyatigorsk	43.89900	43.11100	O	24
Gol’din and Zvonok	2013 [50]	255	*Basilotritus uheni*	Ukraine	Beloskelevaoye	48.45000	39.64000	T	25
Gol’din and Zvonok	2013 [50]	259	*Basilotritus* sp.	Ukraine	Vlasovka	49.30000	33.26700	P	26
Gol’din and Zvonok	2013 [50]	260	*Basilotritus* sp.	Ukraine	Velykaya Andrusovka	49.18300	32.91700	P	27
Gol’din et al.	2014 [51]	269	*Basilotritus sp*.	Ukraine	Nagornoye	49.08300	33.13300	—	—
Gingerich and Zouhri	2015 [24]	280	*Platyosphys aithai*	Morocco	Sabkha de Gueran	25.07667	−13.90763	T	28
Post et al.	2017 [52]	50	Archaeoceti indet.	North Sea	Scheur 10	—	—	—	—
Mychko and Tarasenko	2020 [53]	314	Basilosauridae indet.	Russia	Amber Combine Quarry	54.86700	19.97100	O	**29**
Van Vliet et al.	2020 [54]	124	*Pachycetus robustus*	Germany	Helmstedt and vicinity	—	—	—	—
Davydenko et al.	2021 [55]	70	Basilosauridae incert. sedis	Ukraine	Pyrohiv, Kyiv	50.21000	30.32000	—	—
This study	2022	—	*Antaecetus* sp.	Egypt	Wadi Rayan WR008	29.08000	30.11900	P	30
This study	2022	—	*Antaecetus aithai*	Morocco	El Briej	25.45100	−13.72100	P	31

Locality symbols plotted on the map in Fig 1: *O*, possible Pachycetus (smaller circle); *P*, specimen referred to Pachycetus or a junior synonym (larger circle); *T*, type specimen of a species referred to Pachycetus (star). Label column here gives the number associated with each locality on the map in Fig 1.

### Ukrainian *Zeuglodon paulsonii* of Brandt (1873)

The first specimens of the archaeocete that is now called *Pachycetus* were reported by Afanasii Semenovich Rogovich at the Third Russian Congress of Naturalists in Kyiv in 1871. These were then described in a manuscript by Otto Mikhaĭlovich Paulson that was published by Johann Friedrich Brandt. Brandt included the name *Zeuglodon paulsonii* as a nomen nudem in an abstract [56], and then validated the name in one of many *Anhänge* or insertions in his 1873 monograph on the fossil and subfossil whales of Europe [25]. Paulson’s illustrations were included as the final plate, plate xxxiv, in Brandt’s 1873 monograph. Rogovich was a botanist and Paulson a zoologist at the Russian Imperial University of St. Vladimir in Kyiv, and Brandt was a zoologist in the Academy of Sciences of St. Petersburg.

According to Paulson, in Brandt [25], three vertebral centra and part of a fourth were available in Kyiv. These came from Eocene strata on the right bank of the Tiasmyn River just south of Chyhyryn (Chigirin or Tschigirin), a small city lying 250 km southeast of Kyiv.

The first archaeocete vertebra to be reported from the Donets River basin of Ukraine was published by Leonid Ivanovich Lutugin in 1894. Lutugin [33] reported “a large vertebra representative of the genus *Zeuglodon*” found in glauconitic sandstone near a place he called Pereshchepnoy. This is in Luhansk Oblast.

Alexandre S. Fedorowskij was a Kharkiv University professor studying geology, paleontology, and archaeology. In 1912, Fedorowskij [36] described a more complete set of archaeocete vertebrae, which he identified on page 280 as *Zeuglodon rossicus* = *Z*. *paulsonii*. Fedorowskij excavated three vertebrae in 1909 on the right bank of the south-flowing Donets River near Koropove in Kharkiv Oblast, Ukraine, and farm workers there recovered seven more vertebrae. The age of the glauconitic sand yielding these was thought at the time to be early Oligocene. Fedorowskij regarded the 10 vertebrae as seven lumbars, a sacral, and two anterior caudals, publishing three plates of excellent photographic illustrations. The specimens were deposited in the geological collection of Kharkiv University, but are now lost.

Kellogg [1] proposed the new genus *Platyosphys* for Brandt’s species *Zeuglodon paulsonii*, based largely on Fedorowskij’s specimen, citing the anteroposteriorly long and relatively flat transverse processes on the vertebrae as characteristic of the genus.

In 2001, after a long hiatus, Volodymyr P. Gritsenko named a new species of *Platyosphys*, *P*. *einori*, based on a Ukrainian specimen, TSNU-GM 2638, from Pyrohiv in Kyiv [41]. Gritsenko gave diagnoses for the family Basilosauridae and the genus *Platyosphys*, but no diagnosis for the newly named species. He identified the vertebrae of *P*. *einori* as caudals, and reported their lengths to range from 220 to 150 mm, with transverse processes nearly as long anteroposteriorly as their corresponding centra. Gritsenko compared the pachyostosis of *P*. *einori* to that of Sirenia. Recently Davydenko et al. [55] restudied and reinterpreted TSNU-GM 2638, which they regarded as unidentifiable to genus or species, labeling the specimen “Basilosauridae incertae sedis.” Davydenko et al. identified some of the vertebrae as lumbars. Their descriptions and illustrations indicate that TSNU-GM 2638 is poorly preserved—raising doubt about its perceived distinction from *P*. *paulsonii* (see below)

Evgenij Zvonok [49] described large archaeocete teeth and a large archaeocete rib collected in 2010 and 2011 near Nagornoye in eastern Ukraine. These were identified as “Basilosauridae indet.” The largest of the teeth, upper premolar TSNU-GM 15–2, has a crown measuring 49 × 16 mm in length and width, and the rib, TSNU-GM 15–9, is 560 mm long with a maximum diameter near the distal end of 75 mm. Zvonok also provided a map and a list of sites yielding archaeocetes in eastern Ukraine (Subotiv, Pywycha, Nikopol, Pereshchepyne, Buhaivka, and Luhansk) and southwestern Russia (Pyatigorsk).

Pavel Gol’din and co-authors Zvonok and Tatiana Krakhmal’naya described two vertebrae from Kurenevka in Kyiv, Ukraine, which they identified as “*Eocetus*” sp. [46]. These included a thoracic or lumbar vertebra, NMNH-P OF-1694, and a lumbar vertebra, OF-1695. Both resemble vertebrae previously described as *Platyosphys* [1, 25, 36].

In the following year Gol’din and Zvonok [50] added three new localities south and east of Kyiv: Beloskelevaoye, Vlasovka, and Velykaya Andrusovka. Specimen NMNH-P OF-2096 from Beloskelevaoye was made the type of the new genus and species *Basilotritus uheni*. The type comprises a tympanic bulla, a thoracic vertebra, two thoracic centra, and a rib fragment. KOM 44759 P 201– KOM 44762 P 204 from Vlasovka, four vertebral centra, and KOM 44693 P 195 from Velyka Andrusovka, a vertebral centrum, were identified as *Basilotritus* sp.

The Nagornoye locality yielded specimens, collected in 2004, 2006, 2010, 2011, and 2012, in addition to those described by Zvonok in 2012 [49]. Gol’din et al. [51] studied the new specimens, together with those described earlier, and referred all Nagornoye specimens to “*Basilotritus* sp.” They reported that all of the Nagornoye specimens came from a 40 cm thick interval of shark-rich glauconitic sand. The most informative new specimens were cervical centra with very small vertebrarterial foramina, and a lumbar centrum. It is an open question whether all of the Nagornoye specimens belong to a single basilosaurid.

As Gingerich and Zouhri wrote previously [24]: Gol’din and Zvonok’s separation of *Basilotritus* from *Platyosphys* depended on setting the genus and species *Platyosphys paulsonii* Brandt, 1873, aside as a nomen dubium, in spite of its stated similarity to *Basilotritus uheni*, because “the type specimen is considered to be lost” ([50], p. 263). The validity of a genus and species does not depend on the continued availability of a type specimen, but rather on the indication of a tangible specimen and some description of the morphology involved, whether the specimen itself remains available for study or not. An indication and description were clearly provided by Brandt [25, 56], by Fedorowskij [36], and by Kellogg ([1], p. 97).

### British *Zeuglodon wanklyni* of Seeley (1876)

*Zeuglodon wanklyni* is a species Harry Govier Seeley named in 1876 [27] based on an archaeocete cranium from the Barton Clay at Barton Cliff on the Hampshire coast of southern England. This was evidently a nearly complete cranium when found in 1872, but it was damaged when it was collected. Pieces were salvaged and Seeley made notes on the specimen, which he published four years later. The type is now lost, but the indication and description remain.

The description Seeley [27] gave for the maxillae and maxillary teeth in the type specimen of *Zeuglodon wanklyni* are informative. Kellogg [1] repeated the descriptions, converting Seeley’s measurements to metric units and referring *Z*. *wanklyni* to *Zygorhiza*. Seeley [27] noted (p. 430) that an isolated anterior tooth (canine?) retained a large pulp cavity, which may mean that the specimen was not fully adult. The sizes Seeley and Kellogg gave for measurable teeth of *Z*. *wanklyni* are close to those of deciduous teeth in *Zygorhiza kochii* published by Kellogg [1], but this does not necessarily mean that they were deciduous.

Andrews [34] described an isolated posterior cervical vertebra of *Zeuglodon wanklyni* (NHML-M 11090), that resembles C6 of *Zygorhiza kochii* described by Kellogg [1]. The vertebra was not associated with the skull, but both together suggest that *Zeuglodon wanklyni* was similar in size to *Zygorhiza kochii*. One difference is that C6 of *Zeuglodon wanklyni* has small vertebrarterial foramina (ca. 8 mm in diameter [34]), whereas those of *Zygorhiza kochii* are large by comparison ([1], p. 134).

Halstead and Middleton [39] described a thoracic vertebra of *Zygorhiza wanklyni* from Barton, NHML M-12346, which resembles vertebrae here called *Pachycetus* in having the centrum width substantially greater than the centrum height, in lacking ossified epiphyses, and in having cancellous bone suggesting cartilage where the capitular facets should be. Halstead and Middleton also described another larger thoracic centrum from Barton, NHML M-26552. Finally, NHML M-26553 is an elongated, slightly-flattened, caudal centrum from Barton. The latter two vertebrae were referred to *Basilosaurus*. None of these vertebrae is complete, and one, two, or all three could possibly represent *Pachycetus*.

It is not clear that *Zeuglodon wanklyni* Seeley, 1876, is a species of what is now called *Pachycetus*, but this is possible because *Pachycetus* is known from western Europe during deposition of the classic Bartonian strata at Barton Cliff, and one or more vertebrae from Barton appear referable to *Pachycetus*.

### German *Pachycetus robustus* of Van Beneden (1883)

In 1883 Hanns Bruno Geinitz published the first indication that cetaceans are present in the phosphate beds or *Koprolithenlager* of Helmstedt, Lower Saxony, in north central Germany. Geinitz [57] described a vertebral centrum from Helmstedt, which he considered to be early Oligocene in age. In a later report Geinitz [58] added a second larger centrum and a large rib. The Helmstedt specimens were then sent to the cetacean authority Pierre-Joseph Van Beneden in Leuven for study.

Van Beneden [28] received four vertebral centra and pieces of two or three ribs from Geinitz. These represented cetaceans of two sizes. Van Beneden attributed the first and largest centrum (now MMGD NsT-90), considered a lumbar, and the largest rib (MMGD NsT-92A) to a new genus and species of mysticete, *Pachycetus robustus*, similar in size to the living minke whale *Balaenoptera acutorostrata*. Van Beneden noted that the pedicles of the neural arch were long anteroposteriorly, and the underside of the centrum was distinctive in its flattening and in its furrowed and folded surface. He also noted that the posterior surface of the centrum was substantially larger than the anterior surface. The transverse processes are long anteroposteriorly, but broken near their bases, and it is possible, even likely, that MMGD NsT-90 is a posterior thoracic rather than a lumbar vertebra.

Van Beneden [28] described the rib of *P*. *robustus* as a distal half-rib that measures 450 mm in length, 61 × 56 mm in diameter in the middle, swelling to 80 × 46 mm in diameter near the distal end. He compared the rib to that of a sirenian because of its thickness but confirmed it to be cetacean. The genus name *Pachycetus* was given to acknowledge the thickness of the rib in this larger form, but the vertebral centrum MMGD NsT-90 is the lectotype of *Pachycetus robustus* ([54], p. 123).

Van Beneden [28] described the second and third centra as thoracics, and considered these to represent one species, which was smaller than *Pachycetus robustus*. Both centra lack epiphyses. Van Beneden’s fourth centrum is an even smaller anterior thoracic, measuring 40 × 65 × 55 mm in length, width, and height. Van Beneden was unable to recognize facets for rib heads. This fourth centrum (MMGD NsT-94) is the one later illustrated and designated by Kuhn ([37], Fig 4) as the lectotype of Van Beneden’s *Pachycetus humilis*. Comparison with the pachycetine specimens analyzed here shows that the centrum of *P*. *humilis* is not the shape expected for an anterior thoracic of *P*. *robustus* (see below). Van Beneden [28] did not publish illustrations of either *Pachycetus robustus* or *P*. *humilis*, and the genus and both species were then seemingly forgotten. Van Beneden mentioned *Pachycetus* obscurely in his listing of living and fossil whales in museum collections [32], and Kellogg [1] did not mention *Pachycetus* is his otherwise comprehensive review of Archaeoceti known at the time.

Kuhn [37] redescribed Van Beneden’s specimens of *Pachycetus robustus* and *P*. *humilis* in 1935, illustrated these for the first time, and misinterpreted anterior and posterior in both. Kuhn rejected the name *Pachycetus* as “*uneinheitlich*” or “inconsistent” (whatever he meant by this), and identified Van Beneden’s species as “*Zeuglodon* cf. *isis*” and “*Zeuglodon* sp. indet. cf. *osiris*.” In addition, Kuhn [37] described several new vertebral centra from Trendelbusch, near Helmstedt. The best preserved is a thoracic, lacking epiphyses, that may represent *Pachycetus*. Treue is another German locality near Helmstedt that has yielded *Pachycetus robustus* and possibly *P*. *humilis* ([40], pp. 72–73).

In 2008 Uhen and Berndt [44] described a vertebral centrum from Rohrdorf in Bavaria that they referred to *Eocetus* sp. because of its elongated centrum, anteroposterior elongation of the transverse processes, and the distinctively pockmarked surface of the cortical bone. This was subsequently referred to *Basilotritus* by Gol’din and Zvonok [50] and then to *Pachycetus* by Van Vliet et al. [54]. The premolar illustrated by Uhen and Berndt [44] is probably also a premolar of *Pachycetus*.

Finally, in 2020, Van Vliet et al. [54] clarified the systematic position of *Pachycetus robustus* by adding two lumbar vertebrae, one from Alversdorf (specimen HMS ID20-2/4) and the other from Treue (NMR 9991–13472), both near the type locality of Helmstedt. These are larger and longer than the type specimen of *Pachycetus robustus*, undoubtedly lumbars, and seemingly confirm synonymy of *Pachycetus robustus* and *Pachycetus paulsonii* (see Discussion below).

### Egyptian *Eocetus* (partim) of Stromer (1908)

**Fig 2:**
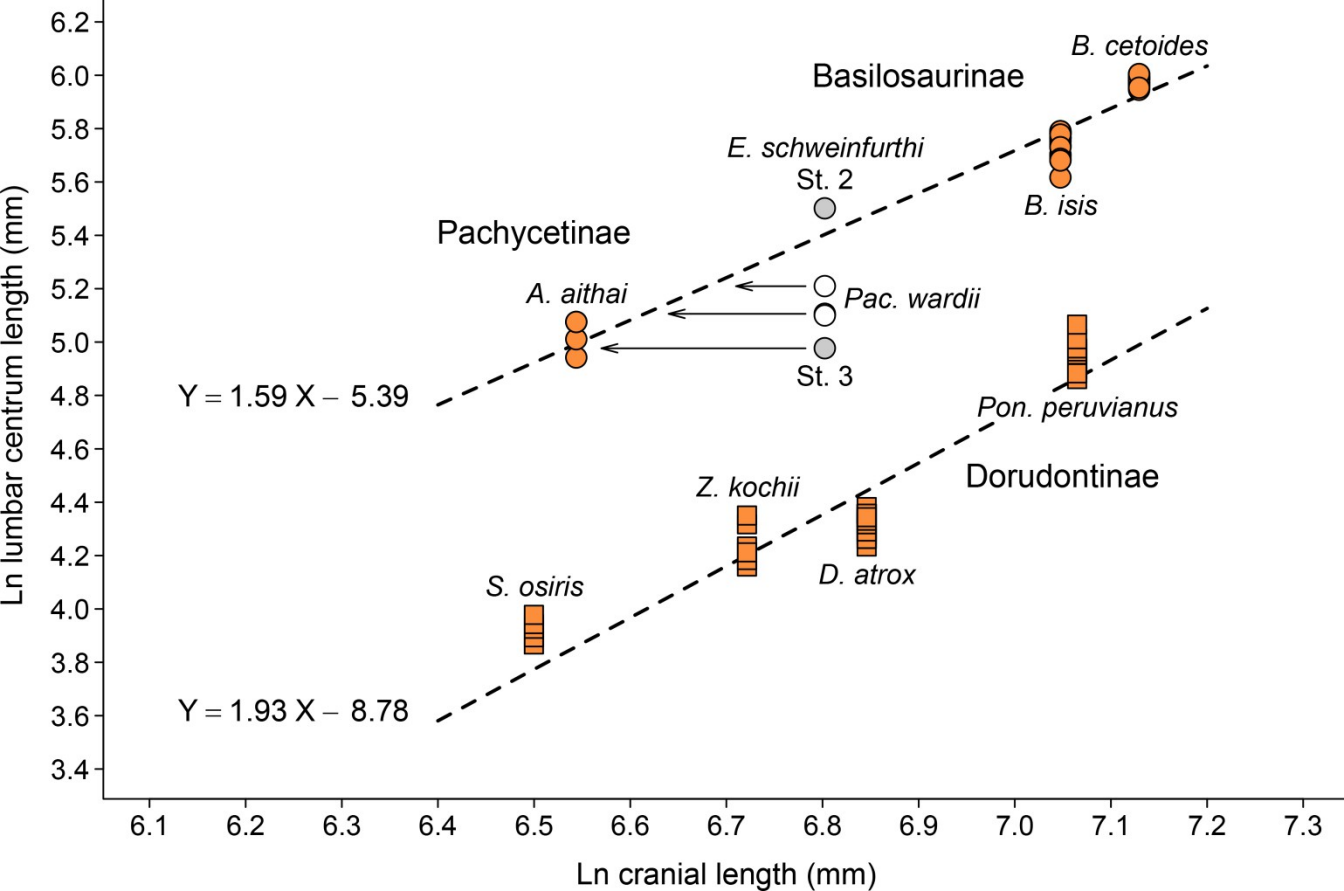
Allocation of lumbar vertebrae to the Egyptian basilosaurid *Eocetus schweinfurthi*. Two subfamilies, Pachycetinae and Basilosaurinae (circles), have long lumbar centra compared to cranial length; one subfamily, Dorudontinae (squares), has short lumbar centra. Lumbar centrum length in St. 2 (SMNS 10934) matches that expected for the skull length of *E*. *schweinfurthi* (SMNS 10986) from the same Bartonian-age strata. The centrum length of St. 3 (10934b) from these strata is shorter than expected. The lumbar centrum of St. 3 has the morphology of a pachycetine, and matches lumbars of *Antaecetus aithai* in size. The cranium of *Pachycetus wardii* is not known but it was probably intermediate in length between those of *A*. *aithai* and *E*. *schweinfurthi*. Cranial and/or lumbar measurements: *Antaecetus aithai*, this study; *Basilosaurus cetoides*, Kellogg [1]; *Basilosaurus isis*, Gingerich et al. (in preparation); *Dorudon atrox*, Uhen [8]; *Eocetus schweinfurthi*, Fraas [13] and Stromer [35, 59]; *Pachycetus wardii*, Uhen [26]; *Pontogeneus peruvianus*, Martínez-Cáceres et al. [9]; *Saghacetus*, Gingerich (in preparation); and *Zygorhiza kochii*, Kellogg [1].

In 1903 Ernst Stromer von Reichenbach described eight archaeocete specimens from Fayum in Egypt, and then when the study went to press added a ninth specimen from high in the Gebel Mokattam stratigraphic section near Cairo ([59], pp. 83–85). The upper Gebel Mokattam interval yielding archaeocetes is now placed in the Giushi Formation and regarded as Bartonian in age [60]. Stromer’s ninth specimen, from Stuttgart, was forwarded for study by Eberhard Fraas. It included two vertebrae, which Stromer called *Wirbel 9a* and *Wirbel 9b*. Stromer’s *Wirbel 9a*, illustrated in his text Fig 1, was described as having a centrum lacking its anterior epiphysis. The centrum, as preserved, measured 245 × 140 × 130 mm in length, width, and height, with a transverse process 155 mm long at its base (making it some 60% of total centrum length). Stromer mentioned that the posterior epiphyseal surface of the centrum was almost flat, nearly circular, and perpendicular to the long axis of the centrum. *Wirbel 9b* was said to be a piece of vertebral diaphysis that was less complete but seemingly larger than the diaphysis of *Wirbel 9a*. In a later study Stromer ([35], p. 109) labeled these large lumbar vertebrae ‘Stuttgart 2’ or, when abbreviated, ‘St. 2’ (now SMNS 10934) and identified them as *Eocetus schweinfurthi*. The genus *Eocetus* and species *E*. *schweinfurthi* were named by Fraas [13, 61] based on ‘St. 1.’ The comparison of vertebral centrum length to skull length in Fig 2 confirms allocation of SMNS 10934 to *Eocetus schweinfurthi*.

In 1908 Stromer ([35], pp. 109–110) referred a third Stuttgart specimen (‘St. 3’) from Gebel Mokattam to *Eocetus schweinfurthi*. This included three vertebrae that Stromer interpreted as not fully grown. One of these, lacking epiphyses, had a centrum complete enough to measure. This centrum, smaller and differently shaped than that of ‘St. 2,’ measured “*über*” 135 × 80 × 65 mm in length, width, and height. Two vertebrae of ‘St. 3’ survive (provisionally numbered SMNS 10934b). Vertebrae of St. 3 (SMNS 10934b) differ from vertebrae of St. 2 (SMNS 10934) in having centra that are smaller and flatter dorsoventrally; having a pachyostotic neural spine, prezygapophyses, and transverse processes; and having anteroposteriorly elongated transverse processes. These are all characteristics of ‘*Zeuglodon*’ *paulsonii* described by Paulson in Brandt [25], *Pachycetus robustus* described by Van Beneden [28], and *Platyosphys aithai* described by Gingerich and Zouhri [24] (now *Antaecetus aithai*, see below).

Stromer ([35], p. 110) indicated that the transverse processes of St. 3 arise from the entire length of the diaphysis, making their length minimally about 90% of total centrum length (as distinct from the 60% relative length of transverse processes calculated for St. 2). The contrasting forms of lumbar vertebrae in *Eocetus* compared to *Pachycetus* and *Antaecetus* are illustrated in Fig 2 of Uhen [26]. *Eocetus* now includes the Bartonian species *E*. *drazindai* formerly placed in Priabonian *Basilosaurus*. *Eocetus* differs from *Basilosaurus* in having large lumbar vertebrae with a longer neural arch and longer transverse processes, both relative to centrum length, but *Eocetus* is closely related and possibly ancestral to *Basilosaurus*.

*Antaecetus aithai* is also known from Bartonian-age strata of Fayum in Egypt. This was found in November of 2008 by a University of Michigan field team working at Wadi Rayan locality WR008. The specimen has not been prepared or cataloged in a museum collection.

### Russian *Zeuglodon paulsonii* of Bogachev (1959)

The first *Pachycetus* specimens from Europe to be found outside Ukraine, Great Britain, and Germany were described by Vladimir Vladimirovich Bogachev from the locality of Khoroshevskaya in the Rostov Oblast of southeastern Russia. Bogachev [38] described four vertebrae identified as *Zeuglodon paulsonii*, all presumably lumbars, based on notes he made in 1940. These were deposited in the Cossack Museum in Novocherkassk.

Later N. P. Kalmykov [47] and A. S. Tesakov et al. [48] described additional archaeocete remains from Khoroshevskaya found by a local resident. Kalmykov identified a tooth interpreted to be M^1^ as *Basilosaurus* sp. The tooth was described as large, but the illustration and measurements are inconsistent, so the size is uncertain. Tesakov et al. described the same specimen as having cervical, thoracic, lumbar, and caudal vertebrae, fragments of limb bones, and a tympanic bulla, but noted that the skull found with the skeleton did not survive. They cited elongation of lumbar centra and transverse processes, vertebral surface texture, and dense layering of cortical bone as characteristic of *Eocetus* (sensu Uhen [26]), and they identified the Khoroshevskaya specimen as *Eocetus*, comparable in size or slightly larger than *Eocetus wardii*.

Another vertebral centrum, KRMHA-KGOM2-13184, was described by Mychko and Tarasenko [53] as a lumbar, but it could possibly be a caudal. Mychko and Tarasenko identified the centrum as Basilosauridae indet.

### American *Eocetus wardii* of Uhen (1999)

Mark D. Uhen [26] recognized and described a distinctive and important archaeocete specimen from North America in the collection of the United States National Museum of Natural History, which he interpreted as a protocetid and named *Eocetus wardii*. This was collected by Lauck W. Ward in 1977 from Laniers Pit near Maple Hill, North Carolina, U.S.A. The type specimen, USNM 310633, includes cranial fragments, four thoracic vertebrae, six lumbar vertebrae, a possible caudal vertebra, a complete rib, and a partial innominate. Complete lumbars range in length from 164 to 183 mm. The innominate is especially interesting because of its distinctive difference from other known basilosaurid innominates (see below).

Later Uhen [42] described a second specimen of *Eocetus wardii*, NCSM 11284, with stylohyals, cervical C7, 12 thoracic vertebrae, and two lumbar vertebrae, sternal elements, ribs, and a scapula. According to Beatty and Geisler [62], this came from the Rocky Point Quarry, near Rocky Point, North Carolina. Centrum lengths for the lumbars from Rocky Point Quarry, both lacking epiphyses, were reported as 134 and 135 mm.

Finally, Weems et al. [45] expanded the geographic range of *Eocetus wardii* by adding two vertebrae from Putneys Mill in Virginia. One of these vertebrae, an anterior thoracic lacking epiphyses (CMM-V-4334), had a centrum length, as preserved, of 63 mm. The other, a posterior lumbar without epiphyses (CMM-V-4335), had a centrum length of 143 mm.

The species *Eocetus wardii* named by Uhen [26] has had an interesting taxonomic history. Geisler et al. [63] questioned attribution to *Eocetus*, and recommended that the species be called *‘Eocetus’ wardii*. Gol’din and Zvonok [50] moved *E*. *wardii* to *Basilotritus* when they named *Basilotritus uheni*. Gingerich and Zouhri [24] referred *E*. *wardii* to *Platyosphys* when they named *Platyosphys aithai*. Finally, Van Vliet et al. [54] synonymized *Basilotritus* and *Platyosphys* with *Pachycetus* and referred *E*. *wardii* to *Pachycetus*.

### North Sea ‘Archaeoceti indet.’ of Post (2007)

In 2007 Klaas Post described three unusual vertebrae recovered by fishermen while trawling across the North Sea bottom off the coast of Belgium and Netherlands [43]. The third of these, NMR 9991–3404, has an elongated lumbar centrum, measuring 190 mm in length without epiphyses. The dorsal surface has a midline crest of bone separating nutrient foramina.

In a later study, Post et al. [52] described a new vertebra, NMR 9991–13472 from ‘Scheur 10’ or channel buoy 10 in the North Sea off the coast of Belgium—which they compared to NMR 9991–3404 and to *Eocetus* sp. of Uhen and Berndt [44]. All are almost certainly specimens of *Pachycetus*.

### Moroccan *Platyosphys aithai* of Gingerich and Zouhri (2015)

*Platyosphys aithai* was named by Gingerich and Zouhri [24] for a series of associated thoracic vertebrae from the Gueran depression in the Sahara southeast of Boujdour, a town on the Atlantic coast of southwest Morocco. Here *P*. *aithai* is placed in a new genus *Antaecetus*. Most thoracic vertebrae of *Pachycetus* and *Antaecetus* have centra that become wider from front to back. Anterior thoracics have roughened surfaces for articulation with the heads of ribs—rather than synovial facets—on both the anterior and posterior sides of the centrum. By the middle of the thoracic series there is an anterior capitular depression but none at the posterior end of the centrum. Some middle thoracics have a slender diapophysis projecting from the centrum for articulation with a rib tubercle. The diapophysis is lost on posterior thoracics, and the rib articulations are open capitular depressions on a projecting parapophyseal surface. Known lumbar vertebrae of *Antaecetus aithai* are rarely complete, but most show the anteroposteriorly long, robust transverse processes extending virtually the entire length of the centrum that are characteristic of *Pachycetus* and *Antaecetus*. Here we describe the first skull and articulated partial skeleton of *Antaecetus* based on a new specimen from Gueran. We also note remains of *Antaecetus* found at the new locality of El Briej.

## Systematic paleontology

The name Archaeoceti was proposed by William Henry Flower for one of three suborders of Cetacea. Flower [64] wrote:

“Among the existing members of the order [Cetacea], there are two very distinct types, the toothed Whales or *Odontoceti*, and the baleen Whales or *Mystacoceti* [Mysticeti], which present as many marked distinguishing structural characters as are found between many other divisions of the Mammalia that are reckoned as orders. As the extinct *Zeuglodon* [*Basilosaurus*], as far as its characters are known, does not fall into either of these groups, but is in some respects an annectent form, I have placed it provisionally, at least, in a third group by itself, named *Archaeoceti*.” ([64], pp. 181–182)

Kellogg [1] enshrined Flower’s ‘third group’ in his *Review of the Archaeoceti*, McKenna and Bell [31] grouped Eocene whales in Archaeoceti, and the name is widely used.

Marx et al. recently wrote in *Cetacean Paleobiology* ([65], p. 2) that “Taxonomically, cetaceans fall into three major groups: ancient whales (archaeocetes), baleen whales (Mysticeti), and toothed whales (Odontoceti).” The name Archaeoceti, whether Latinized or Anglicized, capitalized or decapitalized, has priority and ample precedent for “annectent” whales appearing in the Eocene. Archaeoceti may be paraphyletic in the sense that a member of the group successfully gave rise to a later group (e.g., Odontoceti, Mysticeti, or both), and the same can be said for Basilosauridae. Here Archaeoceti and Basilosauridae are used to represent taxonomic groups in the form and sense of their original authors, with no implication of group sterility (the group had no descendants) nor holophyly (the group includes all of its descendants).

**Mammalia** Linnaeus, 1758 [66]

**Cetacea** Brisson, 1762 [67]

**Archaeoceti** Flower, 1883 [64]

**Basilosauridae** Cope, 1868 [68]

### Diagnosis

Basilosaurids are middle and late Eocene cetaceans that differ from earlier Pakicetidae, Ambulocetidae, and Protocetidae in lacking upper third molars. They also differ in having well-developed pterygoid sinuses that separate left and right middle and inner ears acoustically, short cervical vertebrae, augmented numbers of thoracic and lumbar vertebrae, forelimbs modified into flippers, and hind limbs reduced in size and no longer articulating with the vertebral column. Basilosaurids were the first whales to become fully aquatic.

Basilosaurids differ from later odontocetes and mysticetes in retaining a dental formula of 3.1.4.2 / 3.1.4.3 with recognizable upper and lower incisor, canine, premolar, and molar teeth. Skulls are not telescoped. There is no evidence of baleen. Basilosaurids retain forelimbs with a moveable elbow, and retain hind limbs with reduced but recognizable innominate, femur, tibia, fibula, ankle, and foot bones. Development of a tail fluke is questionable.

**Pachycetinae**, new subfamily

urn:lsid:zoobank.org:act:297935C8-D20F-4339-BD95-193709A240FA

### Type genus

*Pachycetus* Van Beneden, 1883 [28].

### Included genera

*Pachycetus* Van Beneden, 1883 [28], and *Antaecetus*, new genus.

### Diagnosis

Pachycetinae differ from basilosaurine and dorudontine Basilosauridae in several salient features. Pachycetines have pachyosteosclerotic vertebrae not seen in Basilosaurinae or Dorudontinae, and they have pachyosteosclerotic ribs not seen in Dorudontinae. Cartilagenous and ligamentous connective tissue replaces synovial rib articulations. Thoracic vertebrae increase in size from front to back so rapidly that individual centra have a trapezoidal profile. Lumbar vertebrae are elongated like those of Basilosaurinae but differ in having transverse processes nearly as long anteroposteriorly as the centra from which they arise. Most vertebrae have small vascular openings that give surficial bone a distinctively pitted texture. As interpreted here, the innominate of *Pachycetus* differs from innominates associated with basilosaurines and dorudontines in having a much larger obturator foramen.

### Geological age

Pachycetinae have been reported from strata thought to be middle Eocene, late Eocene, and Oligocene in age, but in recent years a consensus has emerged that most or all pachycetines are Bartonian late middle Eocene. The first appearance of Pachycetinae is probably related in some way to global warming of the late Lutetian thermal maximum (LLTM), to the middle Eocene climatic optimum (MECO), or to the short cooler interval between these events. Sea level rise leading to the global high sea stand characteristic of the Bartonian stage/age started in the cool interval between the LLTM and the MECO [7]. The MECO was a Bartonian climate event in the latter part of magnetochron C18r, but the preceding cool interval started in the latest Lutetian in magnetochron C19n [69]. The boundary between magnetochron C19n and C18r, calibrated at 41.0 million years before present, is a candidate for definition of the Lutetian-Bartonian boundary [7].

### Discussion

**Fig 3:**
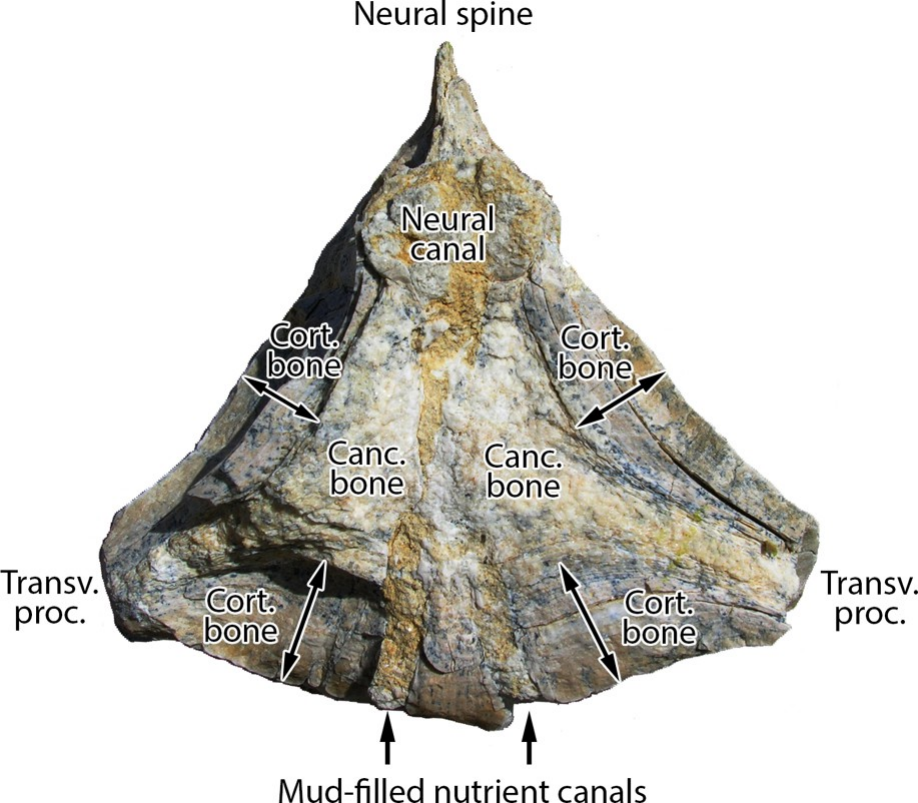
Transverse cross section through a caudal vertebra of *Antaecetus aithai* showing the dense, thickly-laminated, osteosclerotic cortical bone surrounding a central core of cancellous bone. Cortical bone (arrows) is approximately 2 cm thick on the lateral sides of the centrum and 3 cm thick on the ventral side. Cancellous bone (white) is recrystallized, and the neural canal is now filled with crystalline matrix. Mud-filled nutrient canals are stained yellow. This half-centrum is approximately 14 cm wide, left to right, as shown. Specimen photographed in the field at Gueran.

Vertebrae and ribs of Pachycetinae are distinctive in being both *pachyostotic*, thickened with extra layers of lamellar cortical bone (which inspired the name *Pachycetus*), and *osteosclerotic*, having cortical bone that is densely ossified with minimal porosity [70, 71]. The transverse cross section of a pachyosteosclerotic vertebra of *Antaecetus* is shown in Fig 3. Similar cross sections for vertebrae of *Pachycetus* are illustrated by Van Vliet et al. in their plate 3: figs. A2 and B1–B3 [54]. Cones of cancellous bone expand from the center of these vertebrae anteriorly and posteriorly toward the vertebral epiphyses. The surrounding cortical bone is perforated by many small vascular canals, and these give the surface of the vertebra a pitted appearance.

Ribs are pachyostotic and osteosclerotic, with posterior vertebrosternal and anterior vertebrochondrial ribs displaying the most pachyostsis. Rib articulations with thoracic vertebrae have pitted or roughened surfaces in *Antaecetus*, indicating that they were cartilaginous and ligamentous rather than synovial. These articular surfaces are not well described for *Pachycetus*, but where known [26, 50] appear to be pitted or roughened, suggesting that they too were cartilaginous or ligamentous. Lumbar vertebrae of pachycetines are distinctive in having broad anteroposteriorly-elongated transverse processes that approach the length of the centrum.

Pachycetines resemble contemporary basilosaurines and differ from contemporary dorudontines in having elongated trunk vertebrae. However, pachycetines differ from basilosaurines in the pachyosteosclerosis that permeates and envelops their vertebrae. These distinctions have behavioral implications for Pachycetinae (see the Discussion of Locomotion and Behavior below). Phylogenetically, Basilosaurinae and Pachycetinae appear too divergently specialized to have given rise to later whales, leaving generalized Dorudontinae as the group within Basilosauridae most likely to be ancestral to modern Odontoceti and Mysticeti.

The following systematic review summarizes the evidence for recognition of a minimum of three species of Pachycetinae: *Pachycetus paulsonii* in Europe, *Pachycetus wardii* in North America, and *Antaecetus aithai* in Africa.

**Genus *Pachycetus*** Van Beneden, 1883

*Zeuglodon* (in part), Brandt, 1873a [56], p. 112 (nomen nudum). Brandt, 1873b [25], p. 336. Lutugin, 1894 [33], p. 147. Grevé, 1904 [72], p. 67. Fedorowskij, 1912 [36], p. 280. Kuhn, 1935 [37], p. 223. Bogachev, 1959 [38], p. 42.
*Pachycetus* Van Beneden, 1883 [28], p. 32. Lienau, 1984 [40], p. 73. Van Vliet et al., 2020 [54], p. 124.
*Eocetus* (in part), Stromer, 1908 [35], p. 109. Uhen, 1999 [26], p. 514. Uhen, 2001 [42], p. 3. Uhen and Berndt, 2008 [44], p. 57. Weems et al., 2011 [45], p. 273. Gol’din et al., 2012 [46], p. 104. Tesakov et al. [48], 2012, p. 141.
*Platyosphys* Kellogg, 1936 [1], p. 97. Gritsenko, 2001 [41], p. 18. Davydenko et al., 2021 [55], p. 70.
*Basilosaurus* (in part), Halstead and Middleton, 1972 [39], p. 187. Kalmykov, 2012 [47], p. 180.
Archaeoceti indet., Post, 2007 [43], p. 31. Post et al., 2017 [52], p. 50.
Basilosaurinae (in part), Schouten, 2011 [73], p. 19.
*Basilotritus* Gol’din and Zvonok, 2013 [50], p. 255. Gol’din et al., 2014 [51], p. 269.
Basilosauridae (in part), Mychko and Tarasenko, 2020 [53], p. 314.

### Type species

*Pachycetus robustus* Van Beneden, 1883 [28]. Van Vliet et al. [54] designated *P*. *robustus* as the type species of the genus. *P*. *robustus* is the type because it is the species on which *Pachycetus* was based—even though *P*. *robustus* is a junior synonym of the first-named species, *Zeuglodon paulsonii* Brandt, 1873 [25], now included in *Pachycetus*.

### Included species

*Zeuglodon paulsonii* Brandt, 1873 [25] and *Eocetus wardii* Uhen, 1999 [26]. ‘*Zeuglodon*’ *wanklyni* Seeley, 1876 [27], may belong here as an additional species or a synonym (known material is too fragmentary to tell).

### Diagnosis

Differs from *Antaecetus* gen. nov. in being larger and in having more robust teeth with crenulated enamel. The first upper premolar, P^1^, differs from that in *Antaecetus* in being double-rooted or having two fused roots. Posterior upper premolars differ from those of *Antaecetus* in having four rather than three accessory cusps flanking the central cusp anteriorly and posteriorly. Upper molars differ in lacking any distinct medial swelling in the position of the protocone.

### Discussion

In a previous study [24] we stated our reasons for syononymizing *Basilotritus* Gol’din and Zvonok, 2013, with *Platyosphys* Kellogg, 1936. Subsequently, Van Vliet et al. [54] recognized that *Pachycetus* Van Beneden, 1883, is a senior synonym of *Platyosphys* Kellogg, 1936. Thus *Pachycetus* is the appropriate generic name for species formerly included in *Platyosphys* and *Basilotritus*.

There appear to be two valid species of *Pachycetus*, one in Europe (*P*. *paulsonii* Van Beneden, 1883 [28]) and one in North America (*P*. *wardii* Uhen, 1999 [26]). The history of *Pachycetus paulsonii* is complicated and spans 150 years of study, as the following synonymy for the species shows. *Pachycetus wardii* was named more recently and is based on more complete specimens, which has simplified its interpretation.

***Pachycetus paulsonii*** (Brandt, 1873b)

*Zeuglodon cetoides* (in part), Rogovich, 1871 (1873; not seen).
*Zeuglodon paulsonii* Brandt, 1873a [56], p. 112 (nomen nudum). Brandt, 1873b [25], p. 336. Grevé, 1904 [72], p. 67. Fedorowskij, 1912 [36], p. 280, pl. 1: 1–5, pl. 2: 6–10, pl. 3: 11–18. Bogachev, 1959 [38], p. 42.
*Zeuglodon rossicus* Paulson in Brandt, 1873b [25], p. 339, pl. 34: 1–6.
*Pachycetus robustus* Van Beneden 1883 [28], p. 32. Lienau, 1984 [40], p. 73, pl. 9: 13. Van Vliet et al., 2020 [54], p. 124, pl. 2: c1–c4, d1–d2.
*Zeuglodon* cf. *Z*. *isis*, Kuhn, 1935 [37], p. 223, fig. 3a, b.
*Basilosaurus* sp., Halstead and Middleton, 1972 [39], p. 187, fig. 2. Kalmykov, 2012 [47], p. 180, figs. 2–3.
*Platyosphys einori* Gritsenko, 2001 [41], p. 18, fig. 2: 1–5, fig. 3: 1–11.
Archaeoceti indet., Post, 2007 [43], p. 31, figs. 3–4. Post et al., 2017 [52], p. 50, figs. 2–3.
Basilosaurinae (in part), Schouten, 2011 [73], p. 19, figs. of NMR 991–3404.
*Eocetus* sp., Uhen and Berndt, 2008 [44], p. 57, fig. 2. Gol’din et al., 2012 [46], p. 104, figs. 2–4, 5: 2, pl. 1: 1–5, pl. 2: 1–5. Tesakov et al., 2012 [48], p. 141.
Basilosauridae indet., Uhen and Berndt, 2008 [44], p. 59, fig. 4. Zvonok, 2012 [49], p. 87. Mychko and Tarasenko, 2020 [53], p. 314, fig. 1.
*Basilotritus uheni* Gol’din and Zvonok, 2013 [50], p. 255, figs. 2–6.
*Basilotritus* sp., Gol’din and Zvonok, 2013 [50], p. 259. Gol’din et al., 2014 [51], p. 269.
*Pachycetus* sp. indet. A, Van Vliet et al., 2020 [54], p. 126, pl. 1, pl. 2: a1–a3, b1–b3, e1–e2, f1; pl. 3a1–a2, b1–b3, c1–c4, d1–d4.

### Type specimen

One centrum of a thoracic vertebra and two complete centra of lumbars were found at Chyhyryn in Ukraine. These were described by Paulson in Brandt [25]. Paulson named the species involved *Zeuglodon rossicus*, but this is a junior objective synonym of Brandt’s name *Zeuglodon paulsonii*. Kellogg [1] regarded all of Paulson’s vertebrae as co-types, but the holotype is here restricted to the lumbar vertebra retaining both epiphyses and the base of a transverse process (illustrated in Paulson’s fig 2 [25]). According to Gol’din and Zvonok [50], these vertebrae are lost. However, a missing type specimen does not by itself mean a name should be set aside as a *nomen dubium*, and the type specimen of *Zeuglodon paulsonii* (now *Pachycetus paulsonii*) came from a known locality and stratigraphic interval. The type was well illustrated and fully described by Paulson in Brandt [25]. Measurements of the type and other vertebrae of *Pachycetus paulsonii* are listed in Table 6.

### Referred specimens

The principal specimens of *Pachycetus paulsonii* are listed in Table 6. Additional specimens are listed and illustrated in literature cited in the synonymy above.

### Diagnosis

*Pachycetus paulsonii* differs from *Pachycetus wardii* in being significantly larger. Lumbar centra with epiphyses average 266 mm in length for *P*. *paulsonii* and average 171 mm in length for *P*. *wardii*—a difference of 0.44 units on a natural-log scale, which for a linear measurement is equivalent to a difference of about 8 standard deviations.

### Provenance

Known specimens are European, from Germany, Russia, Ukraine, United Kingdom, and the North Sea off the coast of Belgium.

### Geological age

Most or all well-dated specimens come from the Bartonian stage/age of the late middle Eocene.

### Description

The holotype of *Pachycetus paulsonii* is an anteroposteriorly elongated vertebral centrum retaining both epiphyses. This preserves bases of the anteroposteriorly elongated transverse processes and pedicles for a somewhat less elongated neural arch. Paulson in Brandt [25] gave the length, width, and height of the centrum as 260 × 155 × 140 mm, and the holotype was illustrated in plate 34, fig 2, of Brandt [25]. This plate also shows a somewhat shorter, tapering or trapezoidal thoracic centrum, which is notably narrower at the anterior end and wider at the posterior end. A third centrum illustrated on plate 34 is that of a lumbar showing, again, the characteristically elongated transverse processes. Federowskij [36] described and illustrated several lumbar vertebrae of *P*. *paulsonii* and the first-known caudals. Federowskij’s vertebrae are important because of their completeness, preserving neural arches and large flaring metapophyses. Kuhn [37] illustrated the holotype vertebral centrum of *Pachycetus robustus* Van Beneden, 1883 [28], in his fig 3. He interpreted this as a posterior lumbar and compared it to ‘*Zeuglodon*’ *isis*. However, as explained below, it is more likely to be a posterior thoracic of *P*. *paulsonii*. Halstead and Middleton [39] illustrated a tapering or trapezoidal centrum that we interpret as a middle thoracic of *P*. *paulsonii*.

Vertebrae described by Gritsenko [41] are weathered and so poorly illustrated as to be uninterpretable. This was remedied to some extent by Gol’din and Zvonok [50] and Davydenko et al. [55], who showed that vertebrae Gritsenko interpreted as caudals are really lumbars with anteroposteriorly elongated transverse processes. Uhen and Berndt [44] described a premolar and a lumbar vertebra with an elongated centrum, anteroposteriorly elongated transverse processes, and pockmarked bone. Gol’din et al. [46] described and illustrated two thoracolumbar vertebrae similar to those described by Federowskij a century earlier. Kalmykov [47] illustrated a cervical, a thoracic, and a lumbar vertebra. The first well preserved middle thoracic of *P*. *paulsonii* preserving its neural arch was described by Gol’din and Zvonok [50]. The centrum tapers to become wider posteriorly, the neural canal is wide, and the bone of the centrum, neural arch, and neural spine is pachyostotic. Gol’din et al. [51] described cervical vertebrae, which are notable principally for the small size of their vertebrarterial foramina. Van Vliet et al. [54] added a number of cervical, thoracic, lumbar, and caudal vertebrae to *Pachycetus paulsonii* in what they called Morphotype A. Here again cervicals have small vertebrarterial foramina, thoracic centra are tapering, and lumbars have anteroposteriorly long transverse processes.

Most specimens of *Pachycetus paulsonii* are vertebrae, but Van Beneden [28] described the distal half of a large pachyostotic rib. Uhen and Berndt [44] illustrated a premolar. Kalmykov [47] described an upper premolar. Zvonok [49] described a relatively flat mesosternal element, rib pieces, and teeth attributable to *P*. *paulsonii*. Gol’din and Zvonok [50] described a tympanic with the morphology typical of Basilosauridae, and Gol’din et al. [51] added several teeth and a relatively flat xiphisternum. Van Vliet et al. [54] described and illustrated several teeth and pieces of pachyostotic ribs. Teeth of *P*. *paulsonii* are generally similar to those of other Basilosauridae.

***Pachycetus wardii*** (Uhen, 1999)

*Eocetus wardii* Uhen, 1999 [26], p. 514, figs 1.2, 1.4, 3–6. Uhen, 2001 [42], p. 3, figs 1–8. Weems et al., 2011 [45], p. 273, figs 3–4.

### Holotype

USNM 310633, partial skeleton with the rostral fragment of a skull, vertebrae, ribs, and a partial innominate, found at Lanier’s Pit, Maple Hill, North Carolina.

### Referred specimens

CMM V-4334 and 4335, vertebrae. NCSM 11284, partial skeleton with ribs; 11297, vertebrae; 12531, supraoccipital; 13434, vertebral body; 13513, transverse process; 13514, vertebral body; 13676, proximal rib; 13678, partial vertebra; 15663, partial manubrium. USNM 449548, vertebra and ribs.

### Diagnosis

*Pachycetus wardii* differs from *Pachycetus paulsonii* in being significantly smaller. Lumbar centra with epiphyses average 171 mm in length for *P*. *wardii* and average 266 mm in length for *P*. *paulsonii*—a difference of 0.44 units on a natural-log scale, which for a linear measurement is equivalent to a difference of about 8 standard deviations.

### Provenance

Known specimens are North American and come from the states of North Carolina and Virginia in the eastern United States.

### Geological age

The type specimen of *Pachycetus wardii* is from the Comfort Member of the Castle Hayne Formation [26], which could be late Lutetian or early Bartonian in age, near the beginning of the late middle Eocene [74].

### Description

*Pachycetus wardii* from North America is represented by two partial skeletons that are each more complete than any of their European counterparts. The type, USNM 310633, includes an edentulous rostrum with alveoli for large incisors, canines, and a first premolar. Alveoli for the latter, right P^1^, show the tooth to have been double-rooted or to have had two fused roots [26].

USNM 310633 includes four thoracic vertebrae, six lumbar vertebrae, and one vertebra tentatively identified as a caudal [26]. *P*. *wardii* thoracics have centra increasing in size from anterior to posterior. These are tapered, with the posterior width of each notably greater than the anterior width. Thoracic centrum height is substantially less than centrum width. *P*. *wardii* lumbars have centra that change little in size from anterior to posterior. Lumbar centra are more cylindrical in shape than those of thoracics, with anterior and posterior widths being approximately equal. Lumbar centrum height is generally less than centrum width, and the cylindrical shape is thus somewhat flattened dorsoventrally. The caudal is poorly preserved and has never been illustrated. Ribs of *Pachycetus wardii* are generally osteosclerotic and some are pachyostotic with thickened distal ends [26].

USNM 310633 is distinctive in preserving two pieces of an innominate. One piece has a well-formed acetabulum [26], which is normally where the ilium, ischium, and pubis meet before they co-ossify. There is no suggestion of articulation with a vertebral sacrum, and innominates of *Pachycetus wardii* were probably anchored in muscles of the ventral body wall—as they were in *Basilosaurus* and other basilosaurines. A second piece of innominate preserves a portion of the rugose pubic symphysis. We know from comparison of innominates of quadrupedal protocetids with those of fully aquatic dorudontines and basilosaurines that basilosaurids retained a midline pubic symphysis [75] (see Discussion below). However, interpretation of the innominate of *Pachycetus* is complicated because the intervening piece that connected the acetabulum to the symphysis is missing.

The second of the partial skeletons is NCSM 11284 [42] from the Rocky Point Quarry in Rocky Point, North Carolina [62]. This has a well-preserved series of thoracic and lumbar vertebrae, stylohyals, sternebrae, ribs, and a partial scapula.

**Genus *Antaecetus***, new genus

urn:lsid:zoobank.org:act:5F9DDF63-8AE2-4AA6-9698-38840063555A

*Eocetus* (in part), Stromer, 1908 [35], p. 109.
*Platyosphys* (in part), Gingerich and Zouhri, 2015 [24], p. 279.
*Pachycetus* (in part), Vliet et al., 2020 [54], p. 132.

### Type species

*Platyosphys aithai* Gingerich and Zouhri, 2015 [24], p. 280.

### Included species

Type species only, as *Antaecetus aithai*.

### Diagnosis

*Antaecetus* has the distinctive pachyosteosclerotic vertebrae of pachycetine basilosaurids but differs from *Pachycetus* in being smaller and having a notably small cranium. The teeth are more gracile, with smooth rather than crenulated enamel. The first upper premolar, P^1^, differs from that in *Pachycetus* in being single-rooted. Posterior upper premolars differ from those of *Pachycetus* in having three rather than four accessory cusps or denticles flanking the central cusp anteriorly and posteriorly. Upper molars differ in retaining a distinct posteromedial expansion in the position formerly occupied by the protocone.

### Etymology

*N*amed for *Antaios* of Greek mythology (*Antaeus* in Latin, *Anti* in Berber), half-giant son of the sea god Poseidon and earth goddess Gaia; combined with *cetus* (Latin, masc.), whale. According to legend narrated by Plutarch, Antaeus lived in the western desert of North Africa and his tomb was found in what is now Morocco.

### Discussion

*Antaecetus* is known from a skull and much of an associated axial skeleton, both described here. It resembles *Pachycetus*, but differs in being smaller, and in having a relatively small skull and much smaller and more gracile teeth. Larger, more robust teeth of *Pachycetus paulsonii* were described by Uhen and Berndt [44], Kalmykov [47], Zvonok [49], Gol’din and Zvonok [50], Gol’din et al. [51], and Van Vliet et al. [54]. The premaxillae of *Pachycetus wardii* described by Uhen [26] show that it had much larger and more robust incisors than those found in *Antaecetus aithai*.

**Fig 4:**
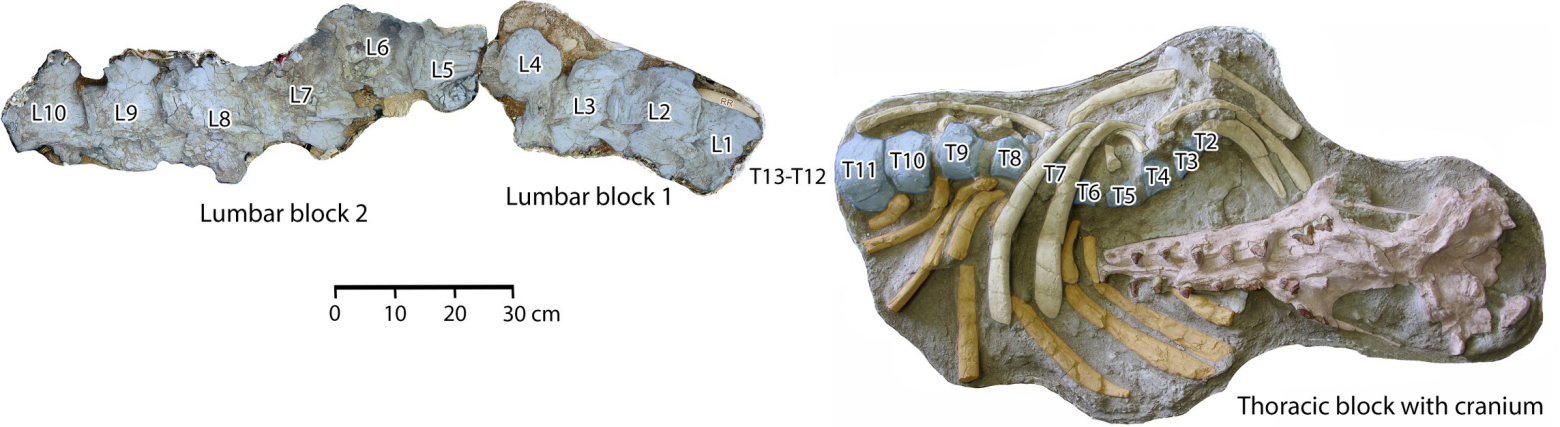
Partial skeleton of *Antaecetus aithai*, specimen FSAC Bouj-200, as collected and preserved in three blocks of sediment. Each block was turned before preparation and then cleaned to expose the underside of the block, which is the side shown here. The cranium is shown in red, teeth in brown, vertebrae in blue, left ribs in orange, and right ribs in yellow. Thoracic vertebrae are numbered from T2 through T11 on the assumption that the skeleton had 13 thoracics. Lumbar vertebrae are numbered from L1 through L10. There may have been more than 13 thoracics, and there may have been more than 10 lumbars.

**Fig 5:**
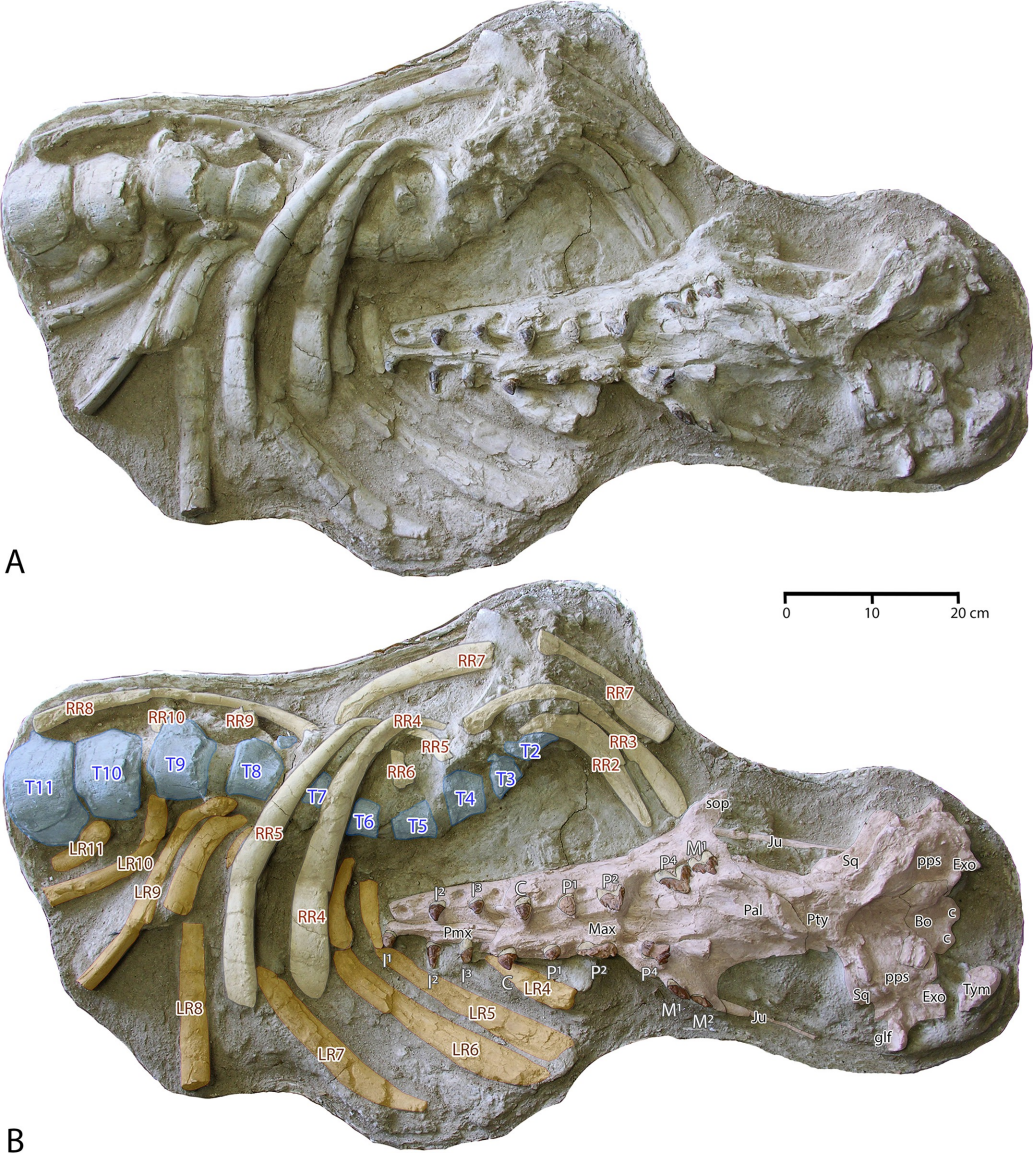
Cranium and thorax of *Antaecetus aithai*, specimen FSAC Bouj-200. The cranium is shown in red and the teeth (labeled I^1^ through M^2^) are shown in brown. Vertebrae are shown in blue, the left ribs in orange, and the right ribs in yellow. Thoracics and ribs are numbered from T2 through T11 and R2 through R11 on the assumption that the skeleton had 13 thoracics and 13 pairs of ribs. Rib numbers correspond to those of matching vertebrae. Abbreviations: *Bo*, basioccipital; *c*, mandibular condyle; *Exo*, exoccipital; *glf*, glenoid fossa; *Ju*, jugal; *Max*, maxilla; *Pal*, palatine; *Pmx*, premaxilla; *pps*, petrosal and surrounding pterygoid sinus; *Pty*, pterygoid; *sop*, supraorbital process of frontal; *Sq*, squamosal; *Tym*, tympanic.

**Fig 6:**
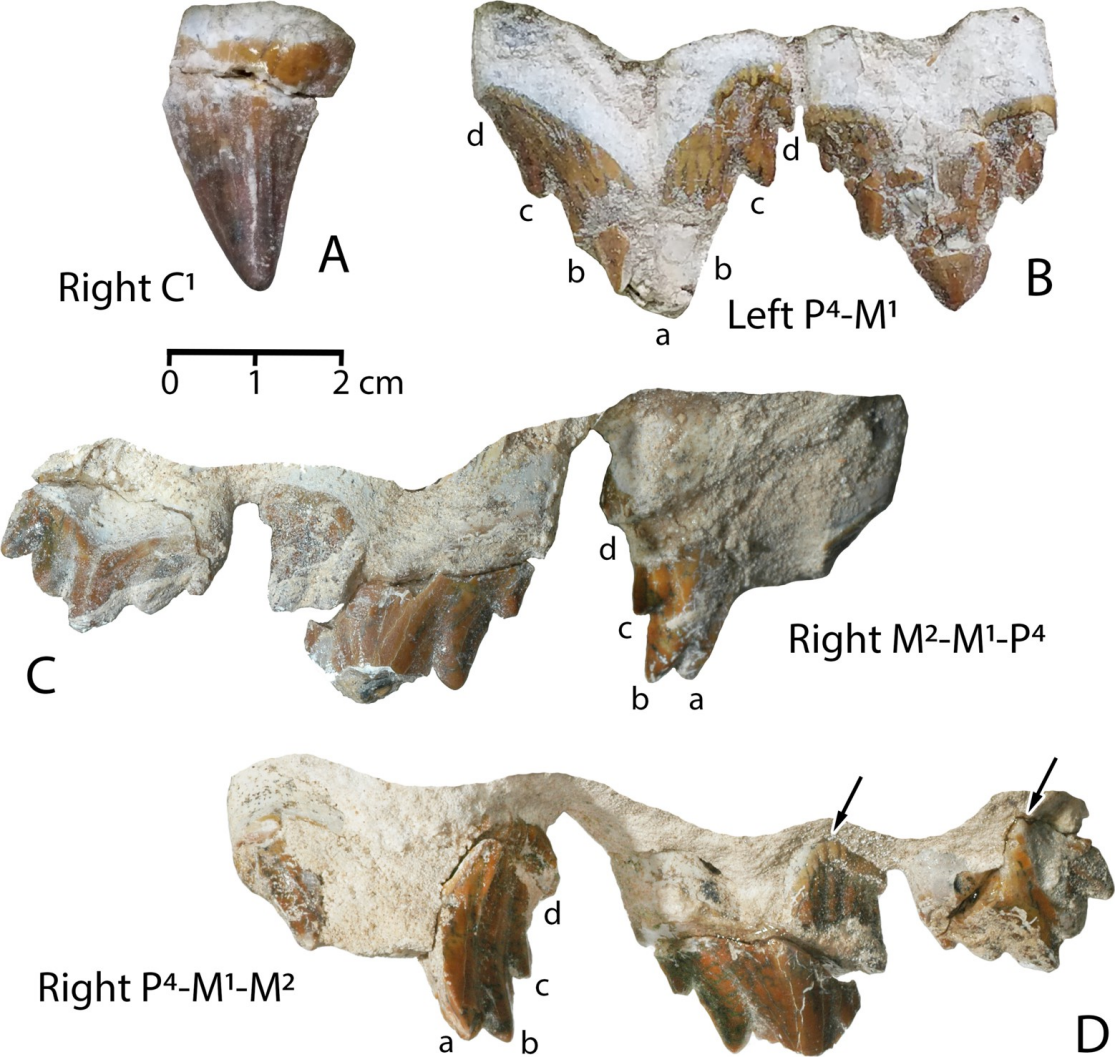
Selected teeth of *Antaecetus aithai*, specimen FSAC Bouj-200. **A**, right upper canine C^1^ in medial view. **B**, left upper P^4^–M^1^ in lateral view. **C**, right upper P^4^–M^2^ in lateral view. **D**, right upper P^4^–M^2^ in medial view. Apical cusps labeled *a* are broken on left and right P^4^; accessory cusps on P^4^ are labeled, *b*, *c*, and *d*. Arrows point to a posteromedial expansion at the base of the crown on the upper molars; this is broader on M^1^ and narrower on M^2^.

**Fig 7:**
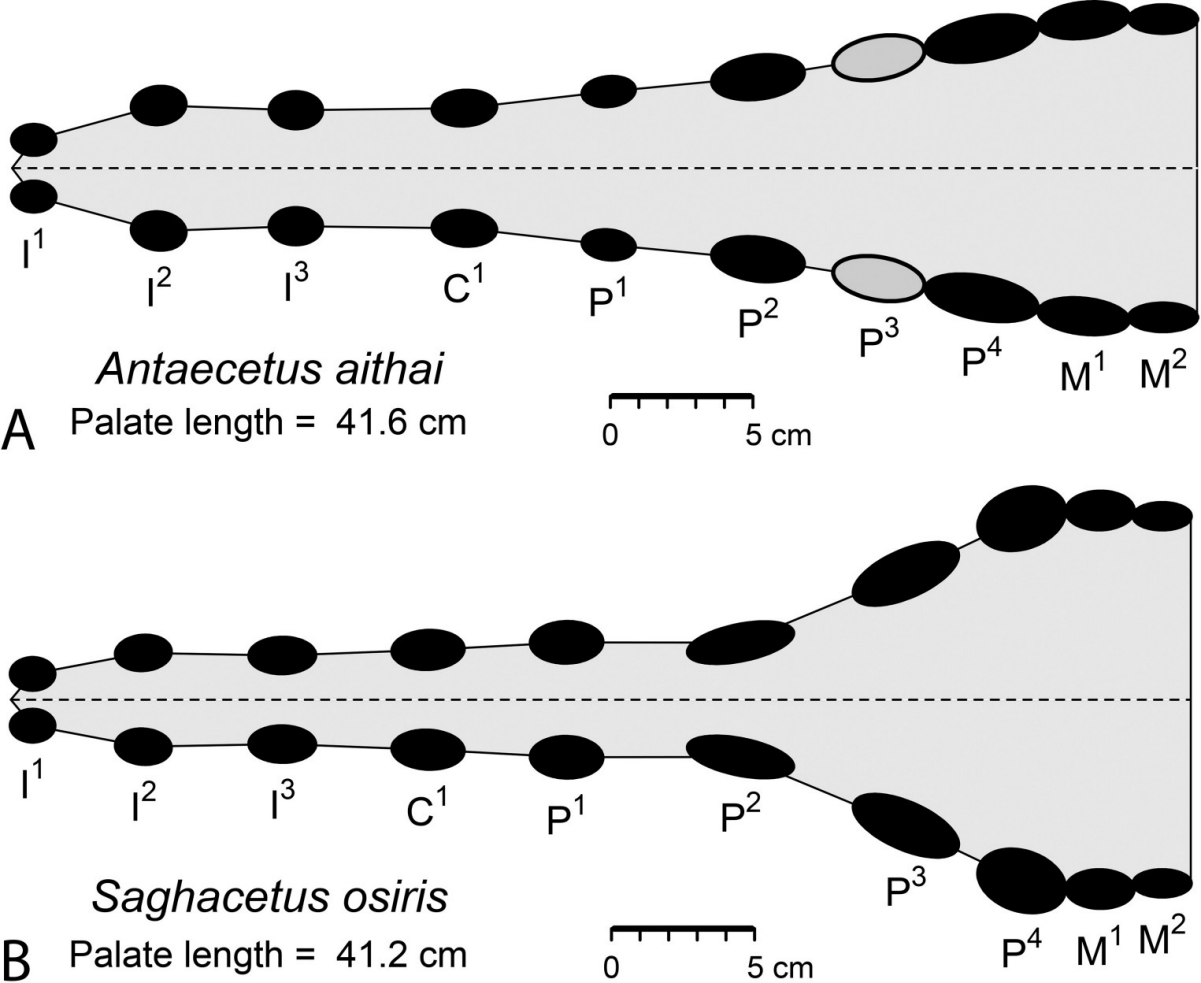
Schematic reconstruction of the palate of *Antaecetus aithai* compared to that of *Saghacetus osiris*. **A**, palatal map for *A*. *aithai* based on FSAC Bouj-200 (Table 3). **B**, palatal map for *S*. *osiris* based on UM 97550. Teeth are shown as ellipses with long axes representing tooth crown lengths and short axes representing tooth crown widths. Spacing is based on diastema length and on the medial-surface-to-midline distance for each tooth. Crown widths for I^2^ and I^3^ are assumed to have the same proportion to crown length as that in I^1^. The missing crown length, width, and height of P^3^ are assumed to be the same as those for P^2^. The diastema preceding P^3^ is arbitrarily assumed to be 10 mm, and the distance from the medial surface of P^3^ to the midline of the palate is assumed to be the average of crown–midline distances for preceding P^2^ and following P^4^. The palate of *A*. *aithai* is approximately the same length as that of *S*. *oriris*, but palatal and tooth shapes differ.

**Fig 8:**
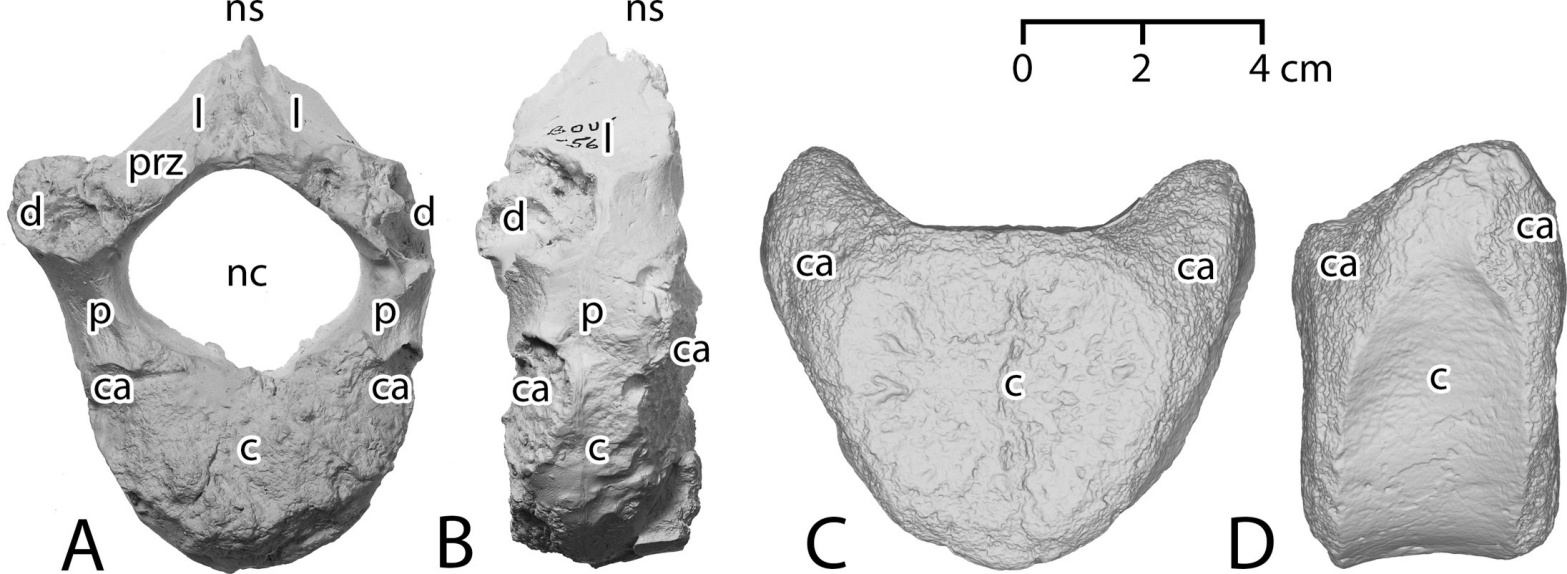
Anterior thoracic vertebrae of *Antaecetus aithai* and ‘*Pachycetus*’ *humilis*. **A**, thoracic T1 of *A*. *aithae*, FSAC Bouj-56, in anterior view; note the reniform centrum and large neural canal. **B**, FSAC Bouj-56 in left lateral view. **C**, centrum of T3 or T4 of ‘*P*.’ *humilis*, MMGD NsT-94, in anterior view; note the larger size and more circular centrum. **D**, MMGD NsT-94 in left lateral view. Illustrations A–B are photographs of a high-fidelity cast, and C–D show a high-resolution 3D digital scan, all reproduced at the same scale. Abbreviations: *c*, centrum; *ca*, capitular articulation; *d*, diapophysis; *l*, lamina; *nc*, neural canal; *ns*, neural spine; *p*, pedicle; *prz*, prezygapophysis.

***Antaecetus aithai*** (Gingerich and Zouhri, 2015)

(Figs 3–6, 7A and 8A–8B)

*Platyosphys aithai* Gingerich and Zouhri, 2015 [24], p. 280.
*Pachycetus aithai*, Van Vliet et al., 2020 [54], p. 124. Davydenko et al., 2021 [55], p. 70.

### Holotype

FSAC Bouj-6, associated thoracic vertebrae. These were first identified as thoracics T1–T4 [24], but comparisons here indicate they are probably T6, T8, and T11-12.

### Referred specimens

FSAC Bouj-7, posterior thoracic vertebra; Bouj-11, three lumbar vertebrae; Bouj-20, partial cranium; Bouj-26, left tympanic bulla; Bouj-200, cranium, thorax, and lumbus described here; measurements of additional specimens are listed in S1 Table.

### Provenance

All known specimens of *Antaecetus aithai* come from the Aridal Formation in the sabkhas of Gueran and El Briej. The new locality of El Briej is located 35 km NNE of Gueran on the map of Gingerich and Zouhri [24].

### Horizon and age

Gingerich and Zouhri [24] and Zouhri et al. [76] summarized the geological context of the Aridal Formation at Gueran. The Aridal Formation is found in the sub-basin of Boujdour, which is part of the larger northeast-southwest trending Tarfaya–La’Youn–Ad-Dakhla depositional basin. The larger basin is the onshore part of a passive continental margin on the Atlantic coast of southwestern Morocco. Ratschiller [77, 78] described the stratigraphic sequence of interest as the Gueran Member of his Samlat Formation. Ratschiller divided this member into: (1) a lower 21 m thick chalk with interbedded siltstones and sandstones yielding shark teeth and other fossils; (2) an upper 22 m thick layer of massive white chalk; and (3) a 2 m thick limestone crust or cap rock. Zouhri et al. ([76], fig 2) published another stratigraphic section on the northeastern flank of the Gueran depression that is shorter than the section of Ratschiller, but described in more detail. The fossiliferous interval at Gueran that yields vertebrate remains is a 1-m-thick, white to light-gray, clayey, silty, poorly-sorted sandstone, with component grains ranging from coarse to very fine in size. This fossiliferous bed is 11 m above the base of the section. Gingerich and Zouhri [24] assigned a Bartonian age to the fossiliferous level at Gueran based on the presence of both protocetid and basilosaurid archaeocetes.

## Description of FSAC Bouj-200

The principal specimen of *Antaecetus aithai* described here is the partial skeleton FSAC Bouj-200 (Fig 4), which includes the cranium and thorax in one 110 × 79 cm block of sediment. An articulated sequence of 10 lumbar vertebrae was collected in two additional blocks. Each block was collected in a plaster jacket. The top surface was weathered by exposure in the field, so each block was turned before preparation. The surface visible now in each jacket is the unweathered side that was originally the bottom surface. The cranium is described first, followed by the dentition, thoracic vertebrae, ribs, lumbar vertebrae, and additional elements.

### Cranium

The cranium of FSAC Bouj-200 is shown in Fig 5, where the palate and basicranium are exposed. These are distorted slightly, so all measurements are necessarily estimates. The anterior edges of the left and right premaxillae (*Pmx*) are preserved, as are the posterior surfaces of both occipital condyles (*occ*). The condylobasal length of the cranium connecting these landmarks is 69.5 cm. The maximum width of the cranium is 31.4 cm, calculated by doubling the distance from the midline of the cranium to its lateral surface just lateral to the right glenoid fossa (*glf*). The premaxillae are elevated slightly relative to the maxillae (*Max*), due to compression, and the maxillae are damaged where the upper third premolars, left and right P^3^, should be (both of these teeth are missing). Bones of the cranium are identified in Fig 5B.

Left and right premaxillae (*Pmx*) each bore three upper incisors (I^1-3^; I^1^ is missing on the left side). The suture between the premaxillae and the left and right maxillae (*Max*) is not visible, but slight displacement and elevation of the premaxillae relative to the maxillae undoubtedly followed the suture. Left and right maxillae each bore seven teeth, of which only the upper canines (C); upper premolars P^1-2^ and P^4^; and upper molars M^1-2^ remain. Upper M^2^ is missing on the left side. A portion of the left supraorbital process (*sop*) of the frontal bone is preserved lateral to and above left M^1^.

The rostral part of the skull is connected to the basicranium by a relatively narrow but robust intertemporal constriction, bordered laterally by large temporal fossae. Palatines (*Pal*) and pterygoids (*Pty*) are the principal elements of this constriction that are visible ventrally, and these are not well preserved. Lateral to the temporal fossae, left and right maxillae are connected to the left and right squamosals (*Sq*) by slender jugals (*Ju*). The squamosals themselves are not well preserved, and the glenoid fossa (*glf*) is only present on the right side of the cranium. The only potions of the basicranium that are identifiable are the basioccipital (*Bo*) with the left and right mandibular condyles (*c*), and the left and right exoccipitals (*Exo*). Left and right petrosals and surrounding pterygoid sinuses (*pps*) are present lateral to the basiocciptal. The petrosals themselves are not well preserved. One tympanic bulla, presumably the right bulla, is preserved floating in matrix just behind the right exoccipital. The tympanic bulla measures approximately 63 × 39 mm in length and width.

Basilosaurid species with good crania include *Basilosaurus cetoides* and *Zygorhiza kochii* described by Kellogg [1], *Dorudon atrox* described by Uhen [8], and *Pontogeneus peruvianus* described by Martínez-Cáceres et al. [9]. All are similar in having a long and relatively narrow rostrum, a broad supraorbital shield, and a broad braincase, with the facial part of the cranium connected to the braincase by a long and relatively narrow intertemporal region. The cranium of *Antaecetus aithai* is smaller and more gracile than crania known for other basilosaurids, but it is otherwise typically basilosaurid in form.

### Dentition

The upper dental formula of *Antaecetus aithai* is 3.1.4.2, as is typical for basilosaurids. Upper incisors in the Bouj-200 cranium (I^1^, I^2^, and I^3^) are all single-rooted teeth with simple, laterally-compressed, conical crowns. Incisors at two positions (I^1^ and I^3^) have crowns whose projecting height is slightly less than their anteroposterior length. Upper second incisors (I^2^) have crowns that are significantly higher and more caniniform than the others, with crowns projecting some 1.3 times higher than their anteroposterior length. All have enamel with narrow ridges and shallow grooves running up the surface, and all have a narrow carina or keel of enamel running up the anterior and posterior edges of the crown. The upper canines (C^1^) are a little larger than the second incisors, but similarly constructed (Fig 6A), with again a single root. The small size of the canine teeth in *A*. *aithai* is worthy of note. Canine dimorphism is little studied in archaeocetes [79], but the small canines of Bouj-200 suggest it is female. Upper incisors and canines are separated from adjacent teeth by diastemata of approximately 25 mm (slight distortions of the cranium mean these cannot be measured precisely).

Three of the four upper premolars are present in each maxilla. P^1^ is a single-rooted tooth. The crown, best preserved on the right side, is smaller but otherwise similar in form to the upper incisors and canines. P^2^ is a double-rooted tooth. The crown is larger than that of any preceding tooth, but it is not well preserved on either side. Posterior to the crown of P^2^, there is a substantial gap where there should be, at most, a very small diastema. This is because P^3^ is missing in Bouj-200. P^4^ is present and double-rooted. The base of the crown of P^4^ is well preserved on the left side in Bouj-200, but the surface enamel is somewhat damaged (Fig 6B). The crown is V-shaped in lateral view, with the dentinoenamel junction descending more or less in parallel with the apex of the crown. P^4^ has a relatively large apical cusp (labeled *a* in Fig 6) flanked anteriorly and posteriorly by three smaller accessory cusps (labeled *b*, *c*, and *d*) decreasing in size away from the apical cusp. The base of the crown is relatively long and narrow like that of other basilosaurids, but it is more gracile than is typical for basilosaurids. P^4^ has a weak cingulum on the lateral side of the crown.

The first upper molar, M^1^, is present in both maxillae of Bouj-200. Left M^1^ is shown in lateral view in Fig 6B. Right M^1^ is shown in lateral and medial views in Fig 6C and 6D. M^1^ is double-rooted. The M^1^ crown has an apical cusp with two smaller but substantial cusps decreasing in size anterior to the apex and two smaller but substantial cusps decreasing in size posterior to it. The posteromedial margin of the crown is expanded slightly posteromedially (arrow in Fig 6D) but there is no protocone cusp. There is a weak cingulum on the lateral side of the M^1^ crown, and a stronger cingulum on the medial side of the crown.

The second upper molar, M^2^, is a simple tooth much smaller than M^1^. This is shown in lateral and medial views in Fig 6C and 6D. M^2^ is double-rooted. The M^2^ crown has an apical cusp and there are again two smaller cusps decreasing in size anterior to the apex and two smaller cusps decreasing in size posterior to it. There is a narrow but distinct medial extension of the medial portion of the crown (arrow in Fig 6D), again with no distinct protocone cusp. The lateral cingulum is weak, and there is seemingly no medial cingulum.

Measurements of the teeth of Bouj-200 are listed in Table 3. This table includes the lengths of diastemata separating successive teeth in the tooth row, and the distance of each tooth from the midline of the cranium. Measurements in Table 3 were used to construct the schematic illustration of the palate shown in Fig 7. The resulting estimate for palate length is 41.6 cm. Thus the length of the palate represents 60% of total skull length. The palate of *Antaecetus aithai* reconstructed in Fig 7 is similar in size to that of *Saghacetus osiris*, which is one of the smallest basilosaurids known.

**Table 3:** Measurements (mm) of teeth and their spacing in the FSAC Bouj-200 cranium of *Antaecetus aithai* from Boujdour in southwestern Morocco.

**Tooth position**	**CL (mm)**	**CW (mm)**	**CH (mm)**	**Diast. (mm)**	**Crown-Mid. (mm)**
**I** ^**1**^	**15.2**	**10.5**	**14.2**	**—**	**4.7**
**I** ^**2**^	**18.9**	**—**	**25.1**	**26.8**	**15.5**
**I** ^**3**^	**18.4**	**—**	**17.6**	**29.5**	**14.0**
**C** ^**1**^	**22.3**	**12.2**	**27.2**	**38.8**	**15.0**
**P** ^**1**^	**18.4**	**10.2**	**19.0**	**30.3**	**21.8**
**P** ^**2**^	**32.4**	**15.1**	**28.5**	**27.0**	**24.4**
**P** ^**3**^	**—**	**—**	**—**	**—**	**—**
**P** ^**4**^	**39.8**	**15.0**	**32.1**	**0.0**	**38.0**
**M** ^**1**^	**31.8**	**12.3**	**25.1**	**0.0**	**45.8**
**M** ^**2**^	**23.9**	**9.7**	**13.5**	**0.0**	**47.5**

Abbreviations: *CL*, mesiodistal crown length; *CW*, mediolateral crown width; *CH*, crown height; *Diast*., diastema separating this from the preceding tooth; *Crown-Mid*., distance from the medial surface of a tooth crown to the midline of the palate.

### Thoracic vertebrae

The ten thoracic vertebrae present in FSAC Bouj-200 are labeled sequentially T2 through T11 in Fig 5B. These numbers were assigned assuming that *A*. *aithai* had 13 thoracic vertebrae, but the total number of thoracics is not known. No cervical vertebrae were found with Bouj-200, and it is likely that the most anterior thoracic is missing in Bouj-200 as well. The missing vertebrae may have been destroyed by erosion, or they may remain in a block of sediment that we were unable to locate. In addition, it appears that two posterior thoracic vertebrae were destroyed during excavation when the block of sediment containing the cranium and thorax was separated from the block containing the anterior lumbars. The only parts of the thoracic vertebrae of Bouj-200 that remain are the articulated centra, and these are only visible in ventral view. Rib facets are not visible. The vertebrae were preserved standing upright when they were buried in sediment in the Eocene, and it appears that the neural arches and neural spines were destroyed by weathering when more recent erosion brought the specimen to the surface.

Following the vertebral numbering sequence in Fig 5, the first vertebral centrum that can be measured in Bouj-200 is that of thoracic T3. Centrum lengths of Bouj-200 are listed in Table 4. Centrum widths and heights cannot be measured accurately because they remain embedded in sediment, but it is clear that thoracics of Bouj-200 have tapered centra. The posterior width for a given centrum exceeds the anterior width of the same centrum, and the anterior width for a given centrum is approximately the posterior width of the preceding centrum. Tapered or trapezoidal centra are characteristic of thoracic vertebrae in Pachycetinae.

**Table 4:** Centrum lengths (mm) of thoracic and lumbar vertebrae in partial skeletons of *Antaecetus aithai* from Boujdour in southwestern Morocco.

Vertebral position	FSAC Bouj-6	FSAC Bouj-7	FSAC Bouj-11	FSAC Bouj-56	FSAC Bouj-200
**T1**	—	—	—	33	—
**T2**	—	—	—	—	—
**T3**	—	—	—	—	39
**T4**	50	—	—	—	45
**T5**	(54)	—	—	—	53
**T6**	57	—	—	—	(56)
**T7**	(60)	—	—	—	(59)
**T8**	(63)	—	—	—	63
**T9**	67	—	—	—	(68)
**T10**	84*	—	—	—	74
**T11**	—	—	—	—	86
**T12**	—	128	—	—	(97)
**T13**	—	—	—	—	(110)
**L1**	—	—	—	—	(124)
**L2**	—	—	—	—	140
**L3**	—	—	—	—	(144)
**L4**	—	—	151	—	(147)
**L5**	—	—	(155)	—	150
**L6**	—	—	158	—	(154)
**L7**	—	—	(163)	—	(157)
**L8**	—	—	(168)	—	160
**L9**	—	—	(172)	—	(160)
**L10**	—	—	175	—	160

Specimens FSAC Bouj-6, 7, and 11 are illustrated in [24]. Numbers in parentheses are interpolated. There is some ambiguity in measurements of Bouj-200 because it is not clear whether vertebral epiphyses are present or absent. Abbreviations: *L*, lumbar; *T*, thoracic. Additional measurements are listed in S1 Table.

Free-standing thoracic centra of *Antaecetus aithai* were illustrated by Gingerich and Zouhri [24], who described FSAC specimen Bouj-6 and mentioned that rib articulations in this species are less an articular facet than a pit or pitted surface. The pits indicate that rib articulations were not synovial like those of basilosaurines and dorudontines, but cartilaginous or ligamentous like those of protosirenid sirenians [80]. Here, after comparison with Bouj-200, we identify the four thoracics included in Bouj-6 as T4, T6, and T9–T10. T10 in Bouj-6 retains a slender diapophysis ([24], fig 8j). Bouj-7 appears to be thoracic T12.

FSAC Bouj-56 (Fig 8A and 8B) is a free-standing first thoracic vertebra, T1, that complements thoracics of Bouj-200 in retaining the neural arch and base of the neural spine. The centrum measures 33 × 56 × 35 mm in length, width, and height. It has reniform anterior and posterior articular surfaces, with a concave dorsal surface that forms the ventral margin of the neural canal. Anterior and posterior surfaces of the centrum expose cancellous bone, and it is not clear whether the epiphyses were ever ossified. The anterolateral surface of the centrum has roughened surfaces for connection to rib capitula at its lateral poles. Capitular depressions are similarly roughened and slightly higher on the posteolateral surface of the centrum. Pedicles are robust, measuring 31 × 13 mm in length and width. These cover virtually the entire anteroposterior length of the centrum. The neural canal is large and almost circular, measuring 40 × 34 mm, with the width being slightly greater than the height. Robust laminae rising from the pedicles converge to enclose the neural canal, forming the dorsal portion of the neural arch. Diapophyses forming the bases for tubercular rib connections project laterally from the left and right pedicle-lamina junctions, but the tubercular rib connections are not preserved. A small prezygapophysis is present on the anterior margin of the right lamina. This is not preserved on the left lamina. The posterior surfaces of the laminae with postzygapophyses are not preserved. The base of the neural spine is small and the spine itself appears to have been gracile and relatively short. T1 of *Antaecetus* is superficially similar to T1 in *Protosiren* [80], but differs in the reniform shape of the centrum, in having a relatively wide neural canal, and in having diapophyses positioned higher on the neural arch.

### Ribs

Thoracic vertebrae, by definition, have ribs associated with them. In FSAC Bouj-200 it is possible to link each of the preserved ribs to its corresponding thoracic vertebra, and the ribs are numbered accordingly (Fig 5). Ribs 4 through 11 are present on the left side of the specimen (ribs LR4, etc.), and ribs 2 through 10 are present on the right side of the specimen (ribs RR2, etc.). Rib heads are not well preserved. It appears from their preservation in Bouj-200 that the most anterior ribs are robust but not notably pachyostotic. Ribs 4, 5, and 6 have a body that is expanded anteroposteriorly near the distal end, making them not only robust but also pachyostotic. Posterior ribs are robust but again do not appear to be pachyostotic. These differences can be seen in Fig 5. The pachyostotic ribs in Bouj-200 have maximum diameters of 47–48 mm.

### Lumbar vertebrae

Ten lumbar vertebrae of FSAC Bouj-200 were recovered in the two blocks of sediment excavated after the skull and thorax were collected. The lumbar blocks are shown in Fig 4 as they were oriented in the field relative to the block containing the skull and thorax. Successive vertebrae in the two blocks are numbered L1 through L10. These are microfractured and slightly deformed in a way that makes preparation difficult. Nevertheless, several features of pachycetine lumbar vertebrae are evident. The lumbars have transverse processes approaching the length of their centra, and many have substantial prezygapophyses or metapophyses extending anteriorly with no corresponding postzygapophyses. The centra of L4 and L5 are oriented in a way that shows paired longitudinal grooves on the ventral surface of the centrum like those illustrated by Fedorowskij [36]. L4 is missing its anterior epiphysis, but enough remains of the diaphysis to show that this was distinctly wider than high. Measurements of lumbar lengths for Bouj-200 are included in Table 4.

The largest lumbar vertebrae of *Antaecetus aithai* at Gueran and El Briej have centra that measure 190 to 195 mm in length, 140 to 145 mm in width, and 120 to 125 mm in centrum height (S1 Table). These are presumably male. Vertebrae of male *A*. *aithai* approach the size of lumbars presumed to be female in contemporary *Eocetus schweinfurthi*, but vertebrae of male *A*. *aithai* are notably smaller than the larger lumbars of male *E*. *schweinfurthi* (see Discussion).

**Fig 9:**
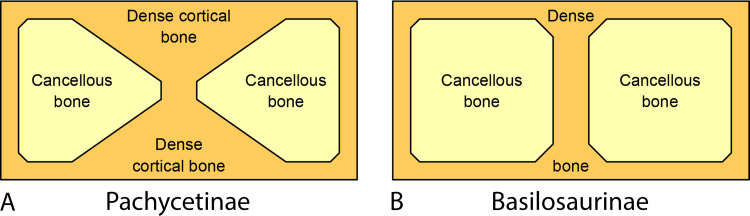
Internal architecture distinguishing the elongated lumbar vertebrae of Pachycetinae and Basilosaurinae. **A**, diagrammatic midsagittal section of the pachycetine *Antaecetus aithai* showing anterior and posterior cones of cancellous bone surrounded by dense cortical bone. **B**, diagrammatic midsagittal section of the basilosaurine *Eocetus schweinfurthi* showing anterior and posterior cylinders of cancellous bone surrounded by dense cortical bone. The proportion of dense cortical bone is much greater in pachycetines than it is in basilosaurines. Drawings are based on midsagittal sections of broken vertebrae observed in the field.

Posterior thoracic, lumbar, and anterior caudal vertebrae of *Antaecetus* and *Eocetus* sometimes break longitudinally to expose a sagittal section of the centrum, and centra of the two genera are notably different in internal architecture (Fig 9). *Antaecetus*, and pachycetines in general, have centra with cones of cancellous bone that flare anteriorly and posteriorly from the center of the centrum, where they are surrounded by a thick outer ring of dense cortical bone. The cortical bone thins toward the anterior and posterior ends of the centrum. This architecture was well illustrated for *Pachycetus* sp. by Van Vliet et al. [54] in their plate 3, figures A1–A2 and B1–B3. In contrast, *Eocetus*, and basilosaurines in general, have cylinders of cancellous bone filling much of the anterior and posterior half of each centrum ([71], fig 14). The centra of posterior thoracic, lumbar, and anterior caudal vertebrae are consequently much denser in pachycetines than they are in the corresponding vertebrae of basilosaurines.

Free-standing lumbar centra of *Antaecetus aithai* were illustrated by Gingerich and Zouhri [24]. Now, after comparison with FSAC Bouj-200, we identify the three centra of Bouj-11 as lumbars L1, L3, and L7.

### Additional elements

There are probably additional skeletal elements of *Antaecetus aithai* present in the FSAC collection. However, based on field observations, there are at least five species of Basilosauridae and three species of Protocetidae at the localities of Gueran and El Briej, with broadly overlapping ranges of body size. Consequently, few isolated elements can be attributed to established taxa with certainty.

In an earlier study [24], we interpreted the partial cranium FSAC Bouj-20 as belonging to *Antaecetus aithai*. Now that there is a cranium of *A*. *aithai* associated with an axial skeleton, it appears more likely that Bouj-20 is a partial cranium of *Eocetus schweinfurthi*. It is too large to represent *A*. *aithai*.

## Discussion

### *Antaecetus aithai* and other Bartonian basilosaurids from Morocco

Most mammals, including whales, have determinate skeletal growth [81, 82], meaning that the skeleton grows ontogenetically until it reachs a definitive size. The most common elements of a mammalian skeleton are vertebrae, which have a range of different pattern-profiles for centrum length plotted against position in the vertebral column. In Basilosauridae, centrum length increases from front to back through the cervical and thoracic vertebrae, reaches a plateau in the lumbar vertebrae, and then decreases in size through the sacral and caudal vertebrae [83, 84]. Determinate growth and the uniformity of lumbar vertebral size means that lumbars are the most useful skeletal elements for recognizing and distinguishing species based on isolated vertebrae.

**Fig 10:**
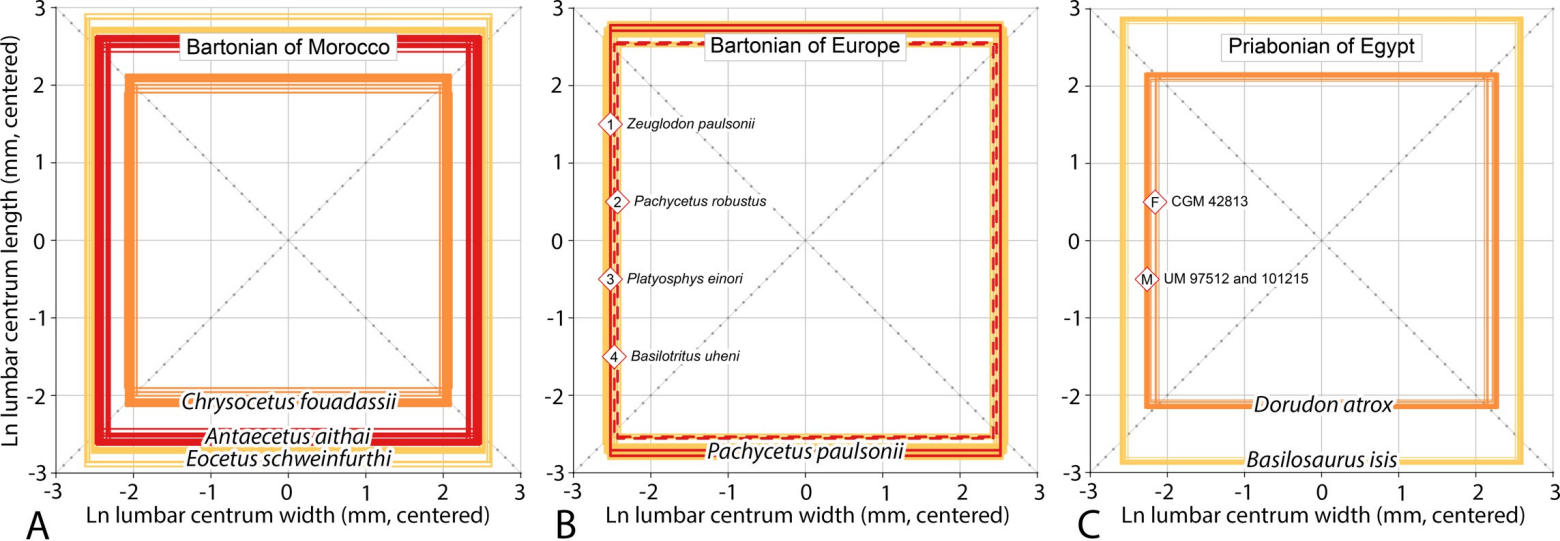
Proportional maps of centrum size and shape comparing lumbar vertebrae of Moroccan Basilosauridae with European *Pachycetus paulsonii* and its synonyms. **A**, lumbar size and shape in the three common basilosaurids from Gueran and El Briej. All are Bartonian late middle Eocene in age. Dorudontine *Chrysocetus fouadassii* is the smallest, with lumbars approximately equal in length and width (based on 27 vertebrae of 8 individual specimens). Basilosaurine *Eocetus schweinfurthi* is the largest, with lumbars longer than they are wide (13 vertebrae of 7 individuals). Pachycetine *Antaecetus aithai* is intermediate in size, with lumbars longer than they are wide (17 vertebrae of 9 individuals). **B**, posterior thoracic and lumbar size and shape for 19 centrum lengths and widths listed in Table 6. These are Bartonian late middle Eocene in age. Lumbar type specimens of *Zeuglodon paulsonii* Brandt, 1873 [25], and *Platyosphys einori* Gritsenko, 2001 [41], are shown with solid red lines. Slightly smaller posterior thoracic type specimens of *Pachycetus robustus* Van Beneden, 1883 [28], and *Basilotritus uheni* Gol’din and Zvonok, 2013 [50], are shown with dashed red lines. All appear to represent a single species, *Pachycetus paulsonii*. **C**, lumbar size and shape in male and female *Dorudon atrox* (CGM 42813 and UM 97512; N = 35) and female *Basilosaurus isis* (CGM 42195; *N* = 19) from Wadi Al Hitan in Egypt are shown for comparison. These are Priabonian late Eocene in age. *D*. *atrox* is smaller, with shorter and relatively wider lumbars [8]. *B*. *isis* is larger, with longer and relatively narrower lumbars (Gingerich et al., in preparation). The variability of the Moroccan taxa is slightly greater than that of Egyptian taxa because more specimens are involved, and measurements of Moroccan taxa were recorded in the field with less measurement precision.

Fig 10A shows a proportional map of lumbar size and shape in the three common late middle Eocene basilosaurid species from Gueran and El Briej in Morocco: the smaller dorudontine *Chrysocetus fouadassii*, the medium-sized pachycetine *Antaecetus aithai* studied here, and the larger basilosaurine *Eocetus schweinfurthi*. Fig 10A was constructed by mapping lumbar centrum length and width for each vertebra of each species as a rectangle on logarithmic axes. The distance between the top and bottom of a rectangle is the natural log of centrum length for that vertebra. Top and bottom lines are plotted equally distant from the center of the graph. The distance between the left and right sides of each rectangle is the natural log of centrum width for the same vertebra, and again the left and right sides are plotted equally distant from the center of the graph. Logarithms standardize variability. The purpose of this representation is a standardized comparison of size and shape, and a standardized comparison of the variation in size and shape: *C*. *fouadassii* has vertebrae smaller than those of *A*. *aithai* and *E*. *schweinfurthi*, but the variability in size and shape is comparable for all three species.

The use of logarithms to compare size in Fig 10A means that the length versus width shapes of the vertebrae are distorted. However, the shapes can still be compared by noting the position of the corners of each rectangle relative to the diagonal lines underlying each plot. Fig 10A shows that *Chrysocetus fouadassii* has lumbars of approximately equal length and width. *Antaecetus aithai* and *Eocetus schweinfurthi* each have lumbars that are notably longer than they are wide. Summary statistics for the three species are listed in Table 5. There are two additional but less common basilosaurids in the Moroccan fauna, not shown here, with lumbar vertebrae wider than they are long. Fig 10A shows how basilosaurid species at Gueran and El Briej can be distinguished based on the size and shape of lumbar vertebrae, even when individual positions within the lumbar series are not known. Any attempted comparison of cervical, thoracic, or caudal vertebrae will only be useful when vertebrae are compared for the same position within the cervical, thoracic, or caudal series.

**Table 5:** Summary statistics for centrum length, width, and height (mm) in lumbar vertebrae of *Chrysocetus fouadassii*, *Antaecetus aithai*, and *Eocetus schweinfurthi* from Gueran and El Briej in southwestern Morocco.

Measurement	N	Minimum (mm)	Maximum (mm)	Mean (mm)	Standard deviation (mm)	Coefficient of variation (std. dev./mean)
***Chrysocetus fouadassii***						
Lumbar length	28	45	72	62.7	8.5	0.14
Lumbar width	27	50	68	61.0	4.6	0.08
Lumbar height	26	43	68	56.5	5.9	0.10
***Pachycetus aithai***						
Lumbar length	28	125	195	165.2	20.4	0.12
Lumbar width	17	100	145	127.1	14.6	0.12
Lumbar height	16	89	125	106.6	13.9	0.13
***Eocetus schweinfurthi***						
Lumbar length	25	200	345	264.0	48.0	0.18
Lumbar width	14	140	190	156.3	15.8	0.10
Lumbar height	14	114	170	132.6	16.4	0.12

Thoracic and lumbar vertebrae of *Pachycetus* from Bartonian age strata of Europe are plotted on the proportional map of Fig 10B (see below for Discussion). For comparison, Fig 10C shows a proportional map of lumbar size and shape in skeletons of two late Eocene basilosaurid species from Egypt: *Dorudon atrox* and *Basilosaurus isis*. *D*. *atrox* has vertebrae smaller than those of *B*. *isis*, and their shapes are different, but the size variation and the shape variation of lumbar centra in the two species are comparable. *Dorudon atrox* has lumbar vertebrae slightly wider than they are long, and the corners of each rectangle fall slightly below the upper diagonals and slightly above the lower diagonals. *Basilosaurus isis* has vertebrae that are notably longer than they are wide, and the corners of each rectangle fall well above the upper diagonals and well below the lower diagonals. The *D*. *atrox* rectangles in Fig 10C include 10 lumbar vertebrae of one specimen (CGM 42813) identified as female, and 25 lumbars of two specimens (UM 97512 and 101215) identified as male. The *B*. *isis* rectangles in Fig 10C include 19 lumbar vertebrae of one specimen, CGM 42195, identified as female.

### Length profile of *Antaecetus aithai* vertebrae

**Fig 11:**
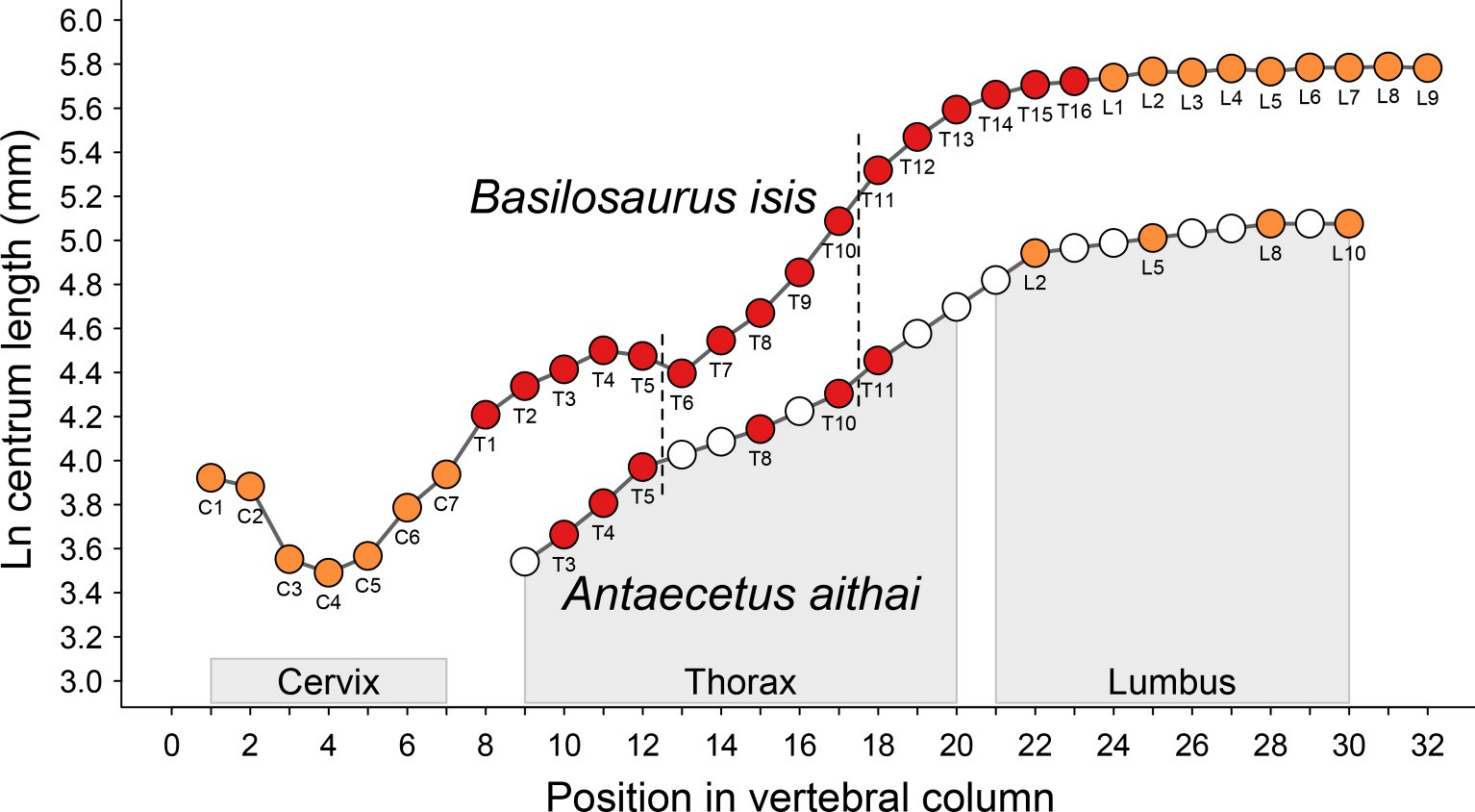
Profile of vertebral centrum length in the FSAC Bouj-200 partial skeleton of *Antaecetus aithai* compared to the profile for Basilosaurus *isis* (CGM 42195). Thoracic vertebrae are shown in red, cervicals and lumbars in orange. Open symbols are interpolated. Dashed lines approximate the inflection points that separate anterior thoracics with fixed vertebrosternal ribs from middle thoracics with more mobile vertebrochondral ribs, and separate middle thoracics from posterior thoracics with floating ribs. Centrum lengths of *A*. *aithai* are approximate because these cannot be measured precisely. *A*. *aithai* is shown as having 13 thoracic vertebrae, but the number may have been greater.

The vertebral centrum lengths for the FSAC Bouj-200 partial skeleton of *Antaecetus aithai* (Table 4) are plotted against their position in the vertebral column in Fig 11. Corresponding centrum lengths for the CGM 42195 skeleton of *Basilosaurus isis* are used as a model. We assume that *Antaecetus aithai* had the same number of cervical vertebrae, seven, as in all other archaeocetes, but the number of thoracics and lumbars in the vertebral column of *A*. *aithai* is not known. The minimum number of thoracics in other archaeocetes is 13 and the number of lumbars in Bouj-200 is 10, which we take as minima for both the thoracic and lumbar series of *A*. *aithai*. Two additional landmarks are available: the first is the inflection point where anterior thoracic lengths increase more slowly (or decrease) and middle thoracic lengths increase more rapidly, and the second is the inflection point where middle thoracic lengths increase more rapidly and posterior thoracic lengths increase more slowly (vertical dashed lines in Fig 11). We take these landmarks to correspond approximately to the points that separate fixed anterior thoracic vertebrae from more-mobile middle and posterior thoracics. Fixed anterior thoracic vertebrae have vertebrosternal ribs that connect each vertebra through its ribs and short costal cartilages directly to the sternum. More-mobile middle thoracic vertebrae have vertebrochondral ribs connecting each vertebra through its ribs and long costal cartilages to the distal ziphisternum. Finally, posterior thoracic vertebrae have floating ribs that are not constrained by costal-cartilage connections to the sternum.

With these assumptions, we infer that *Antaecetus aithai* may have had as few as six anterior thoracic vertebrae (as found in the basilosaurine *Basilosaurus isis* and the dorudontines *Dorudon atrox* and *Pontogeneus peruvianus*). *A*. *aithai* may have had as few as 7 middle and posterior thoracic vertebrae (many fewer than those in *B*. *isis* or *D*. *atrox* and *P*. *peruvianus*; see, e.g., [8] p. 83, and [9] p. 17). We assume that two posterior thoracics of *A*. *aithai* were destroyed when the skeleton was cut in the field to facilitate collection of the skull and thorax, however if three or more thoracics were destroyed during collection, then *A*. *aithai* had more than 13 thoracics. Ten centra were recovered when the lumbar vertebrae were collected, but this is a minimum number and there may have been more lumbars.

### Synonymy of European *Pachycetus*

Lumbar vertebrae of *Pachycetus* from Bartonian age strata of Europe are listed in Table 6, with literature sources and measurements of centrum length, width, and height. These are plotted on the proportional map of Fig 10B for comparison with Moroccan and Egptian forms. Seventeen different lumbar vertebrae are plotted in yellow in the background of Fig 10B. Two of these are type specimens highlighted with solid red lines: the type of *Zeuglodon paulsonii* Brandt, 1873 [25] (diamond 1) and the type of *Platyosphys einori* Gritsenko, 2001 [41] (diamond 3). Two posterior thoracic vertebrae are plotted in yellow in the background of Fig 10B. Both are type specimens, highlighted with dashed red lines to distinguish them from type specimens based on lumbars: the type of *Pachycetus robustus* Van Beneden, 1883 [28] (diamond 2) and the type of *Basilotritus uheni* Gol’din and Zvonok, 2013 [50] (diamond 4).

**Table 6:** Centrum measurements for posterior thoracic and lumbar vertebrae of European *Pachycetus paulsonii* and its synonyms.

Author	Genus and species	Locality	Specimen	Pos.	Length	Ant. wid.	Ant. hgt.	Post. wid.	Post. hgt.	Epiphyses
**Brandt, 1873 [25]: 339**	***Zeuglodon paulsonii***	**Chyhyryn**	**Lost**	**L**	**260**	**155**	**140**	**155**	**140**	**Present**
Brandt, 1873 [25]: 339	*Zeuglodon paulsonii*	Chyhyryn	Lost	L	228	150	145	150	145	Missing
**Van Beneden, 1883 [28]: 28**	***Pachycetus robustus***	**Helmstedt**	**MMGD NsT-90**	**T**	**160**	**120**	**100**	**120**	**100**	**Missing**
Fedorowskij, 1912 [36]: 260	Zeuglodon paulsonii	Khoropove	Lost	L	283	161	151	161	151	Present
Fedorowskij, 1912 [36]: 260	Zeuglodon paulsonii	Khoropove	Lost	L	282	165	157	165	157	Present
Fedorowskij, 1912 [36]: 260	Zeuglodon paulsonii	Khoropove	Lost	L	274	164	157	164	157	Present
Fedorowskij, 1912 [36]: 260	Zeuglodon paulsonii	Khoropove	Lost	L	231	157	147	157	147	Present
Kuhn, 1935 [37]: 224	*Pachycetus robustus*	Helmstedt	MMGD NsT-90	T	150	120	95	110	95	Missing
Bogachev, 1959 [38]: 41	*Zeuglodon paulsonii*	Khoroshevskaya	CMN	L	250	170	150	140	150	—
Bogachev, 1959 [38]: 41	*Zeuglodon paulsonii*	Khoroshevskaya	CMN	L	240	180	150	160	145	—
Bogachev, 1959 [38]: 41	*Zeuglodon paulsonii*	Khoroshevskaya	CMN	L	225	175	160	160	160	—
Gritsenko, 2001 [41]: 18*	***Platyosphys einori***	**Pyrohiv, Kyiv**	**TSNU-GM 2638**	**L**	**225**	**155**	**—**	**155**	**—**	**Missing**
Gritsenko, 2001 [41]: 18*	*Platyosphys einori*	Pyrohiv, Kyiv	TSNU-GM 2638	L	212	153	—	140	—	Missing
Uhen and Berndt, 2008 [44]: 57	*Eocetus* sp.	Rohrdorf	Berndt collection	L	210	161	—	161	—	Missing
Gol’din et al., 2012 [46]: 111	*"Eocetus"* sp.	Kurenevka	NMNH-P OF-1695	L	160	147	120	158	134	Missing
**Goldin and Zvonok, 2013 [50]: 255**	***Basilotritus uheni***	**Beloskelevatoye**	**NMNH-P OF-2096**	**T**	**159**	**98**	**78**	**140**	**80**	**—**
Goldin and Zvonok, 2013 [50]: 260	*Basilotritus* sp.	Velykaya Andrusovka	KOM 44693 P 195	L	202	136	126	157	125	Missing
Gol’din et al., 2014 [51]: 271	*Basilotritus* sp.	Nagornoye	NMNH-P Ngr-12	L	193	186	124	186	124	Missing
Van Vliet et al., 2020 [54], p. 145	*Pachycetus robustus*	Helmstedt	MMGD NsT-90	T	166	—	—	129	96	Missing
Van Vliet et al., 2020 [54], p. 146	*Pachycetus* sp.	Alversdorf	ID20-2	L	239	130	140	131	124	Missing
Van Vliet et al., 2020 [54], p. 146	*Pachycetus* sp.	Treue	NMR 9991–51759	L	225	140	178	—	130	Missing

Type specimens are in boldface type. Most vertebrae are lumbars (L), but MMGD NsT-90 and NMNH-P OF-2096 are posterior thoracics (T). *Measurements and interpretations of vertebrae described by Gritsenko [41] follow Davydenko et al. [55].

**Fig 12:**
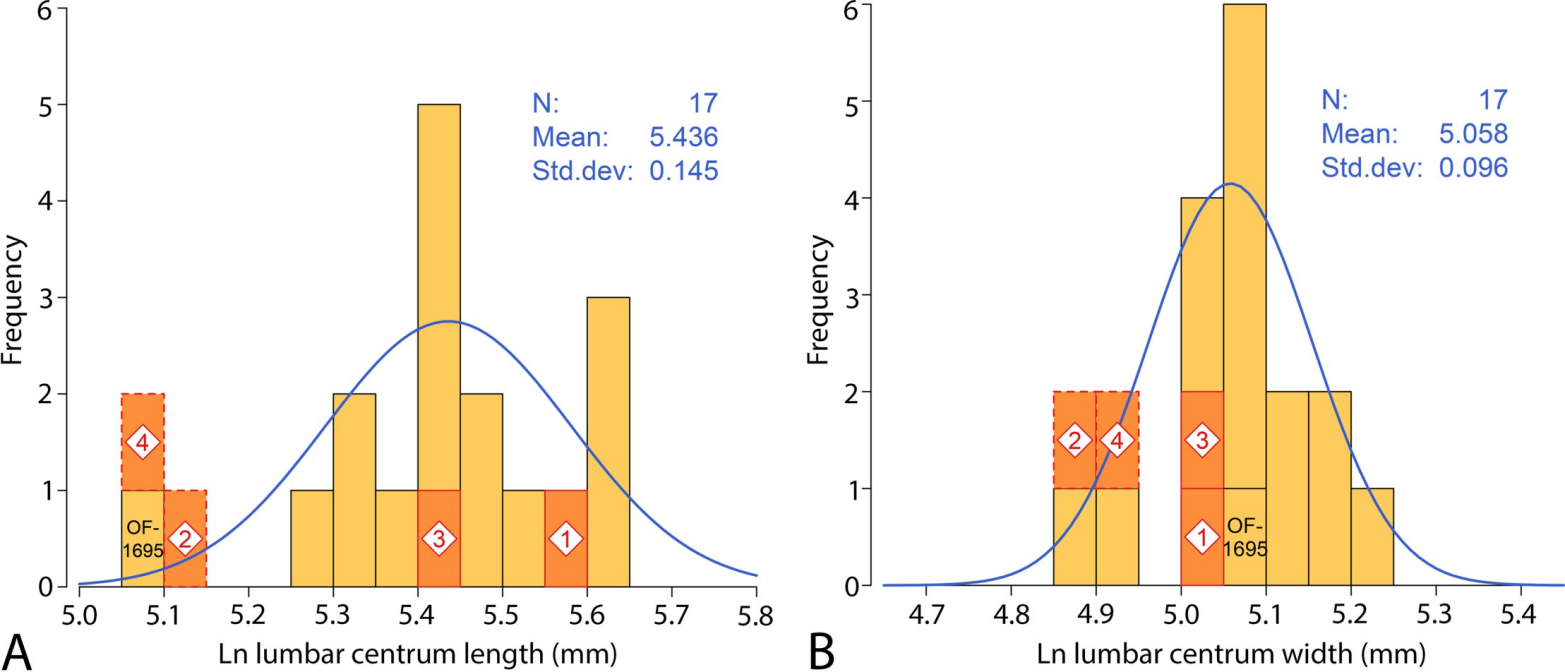
Variability of lumbar length and width in European *Pachycetus paulsonii* and its synonyms. **A**, histogram of centrum length for 17 lumbar vertebrae listed in Table 6. Type specimens of *Zeuglodon paulsonii* Brandt, 1873 [25], and *Platyosphys einori* Gritsenko, 2001 [41], in this sample are labeled with diamonds numbered 1 and 3. Slightly smaller posterior thoracic type specimens of *Pachycetus robustus* Van Beneden, 1883 [28], and *Basilotritus uheni* Gol’din and Zvonok, 2013 [50], are labeled with diamonds numbered 2 and 4. OF-1695 is a short-centrum lumbar lacking epiphyses. **B**, histogram of centrum width for the 17 lumbar vertebrae plotted in panel A. Posterior-thoracic type specimens are shown as well (diamonds 2 and 4). All appear to represent the species *Pachycetus paulsonii*.

Length and width measurements for the 17 lumbar vertebrae in Table 6 are displayed as histograms in Fig 12. The histogram for lumbar length (Fig 12A) spans a range of 0.6 natural log units (5.05 to 5.65 ln units), with a calculated standard deviation of 0.145. This is equivalent to a coefficient of variation of 14.5% for the raw measurements. The histogram for lumbar width (Fig 12B) spans a range of 0.4 natural log units (4.85 to 5.25 ln units), with a calculated standard deviation of 0.096. This is equivalent to a coefficient of variation of 9.6% for the raw measurements. The greater variability of lumbar length is due to variability in the presence of epiphyses, and to the independent variability of diaphysis length (the standard deviation for nine lumbars lacking epiphyses is 0.120).

We cannot be certain that all of the lumbar vertebrae listed in Table 6 represent a single species, but close clustering of their measurements in the histograms of Fig 12 makes this plausible. We interpret all to represent *Pachycetus paulsonii*. The two type specimens that are most different are those representing the two species, *Pachycetus robustus* and *Basilotritus uheni*, which are based on posterior thoracic centra. These are relatively short because they are thoracics, but each is as wide as some lumbar vertebrae of *P*. *paulsonii*. It will not be surprising if additional archaeocete genera and species are found to be present in Bartonian age strata of Europe, but this has not yet been demonstrated.

### Systematic position of *Pachycetus humilis*

Van Beneden [28] named the genus *Pachycetus* and its type species *Pachycetus robustus* based on what he called a lumbar centrum, MMGD NsT-90, from the phosphate beds at Helmstedt in Germany. MMGD NsT-90 is probably a posterior thoracic of *Pachycetus paulsonii* [54]. Van Beneden [28] named a smaller archaeocete *Pachycetus humilis* from the same phosphate beds. The type specimen of *P*. *humilis*, MMGD NsT-94, is illustrated in Fig 8C and 8D, where it is compared to FSAC Bouj-56 described here. The NsT-94 centrum, lacking epiphyses, is larger, measuring 40 × 65 × 55 mm in length, width, and height [28]. Pachycetine thoracics increase markedly in size from front to back (Fig 11), and based on size alone the NsT-94 centrum might be considered an anterior thoracic vertebra conspecific with *P*. *robustus*. However, the centrum height and the more circular centrum outline of NsT-94 contrast with the broader, shallower vertebral centra of pachycetine thoracics [24, 26, 42, 50]. Thus we agree with Van Vliet et al. [54] that NsT-94 and ‘*Pachycetus*’ *humilis* are likely to represent a second Helmstedt archaeocete distinct from *Pachycetus*.

### Interpretation of the innominate of *Pachycetus wardii*

**Fig 13:**
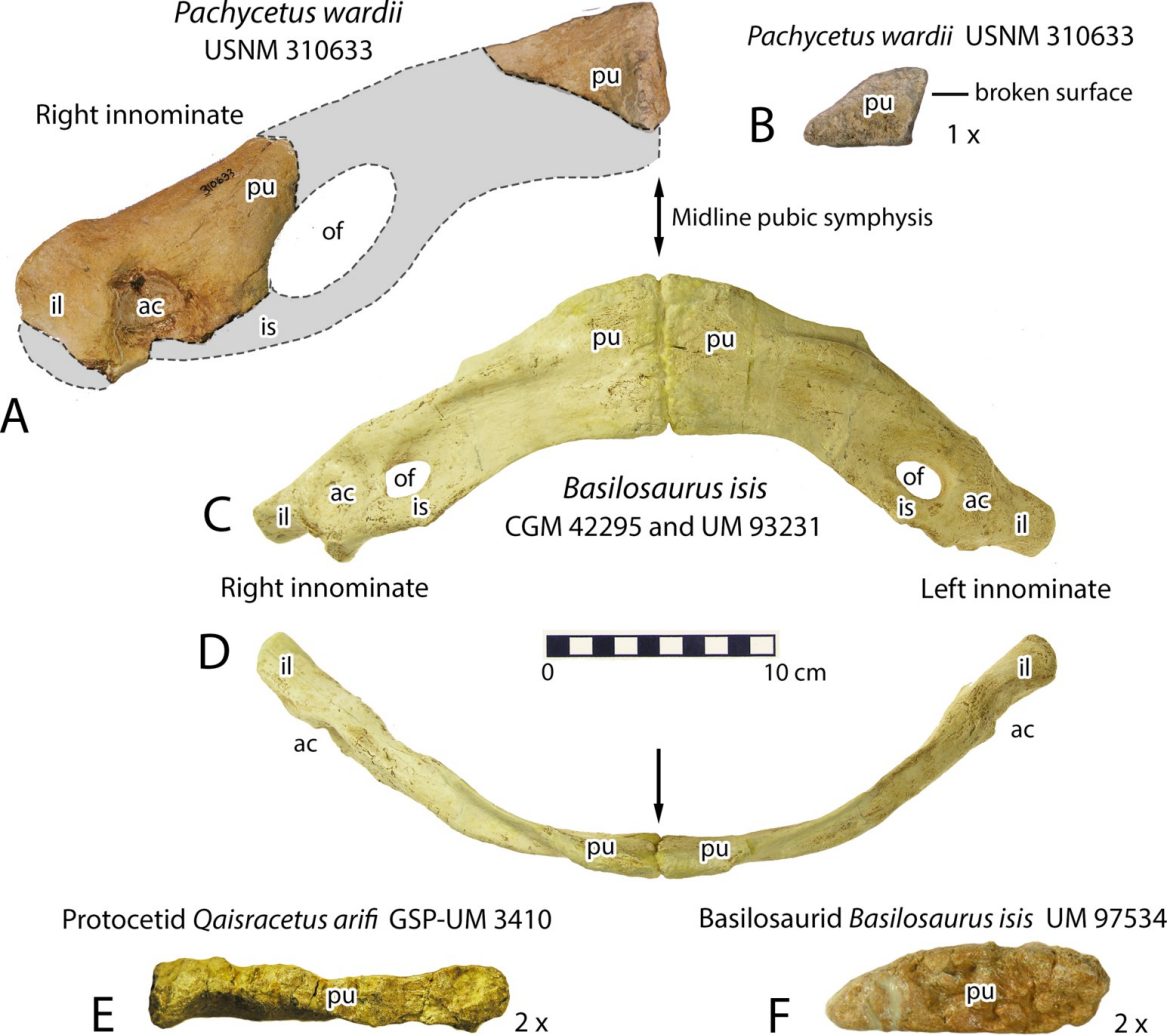
Innominate of *Pachycetus wardii* compared to those of *Basilosaurus isis*. **A**, right innominate of *P*. *wardii*, USNM 310633, in ventral view (anterior at the top). Two pieces are preserved, but the shape of bone connecting these, in gray, is conjectural, as are the length and width of the innominate as a whole. **B**, pubic symphysis of *Pachycetus wardii*, USNM 316033, in medial view. **C–D**, pelvic model for *B*. *isis* based on right and left innominates, CGM 42295 and UM 93231, in ventral view (anterior at the top) and in anterior view. **E**, pubic symphysis of the protocetid *Qaisracetus arifi*, GSP-UM 3410 [88] in medial view. **F**, pubic symphysis of the basilosaurid *Basilosaurus isis*, UM 97534, in medial view. Articular surfaces for all three symphyses are similarly rugose. GSP-UM 3410 and UM 97534 are shown at twice the scale of the other images. Abbreviations: *ac*, acetabulum; *il*, ilium; *is*, ischium; *of*, obturator foramen; *pu*, pubis.

Relatively complete innominates are known for the basilosaurines *Basilosaurus cetoides* [85] and *Basilosaurus isis* [75], for the dorudontines *Chrysocetus healyorum* [21] and *Dorudon atrox* (unpublished), and for two transitional early mysticetes [86, 87]. A partial innominate is known for the dorudontine *Pontogeneus peruvianus* [9]. Basilosaurine and dorudontine innominates are similar in having a relatively small acetabulum (frequently injured and remodeled in life); a small, oval obturator foramen; and a prominent midline symphysis (Fig 13).

Uhen [26] described a partial innominate of *Pachycetus wardii* (Fig 13A) which is part of the type specimen USNM 310633. He placed the species initially in *Eocetus*, at a time when *Eocetus* was regarded as a protocetid, recognizing that the innominate of *P*. *wardii* was different from those of known protocetids. The only landmark that can be identified with certainty on the partial innominate described by Uhen is the acetabulum. Here we add a second piece of innominate, a portion of the pubic symphysis (Fig 13A), which was found among bone remnants of USNM 310633. The two pieces, acetabular and symphyseal, may be parts of the same innominate, but they do not contact. Consequently, the outline of the *P*. *wardii* innominate in Fig 13 is still uncertain.

The ramus and symphysis of the *Pachycetus wardii* innominate are compared to complete left and right innominates of *Basilosaurus isis* in Fig 13. Landmarks identifiable in both species are the acetabulum for articulation with the head of the femur, and the midline pubic symphysis (arrows) for articulation with the opposite innominate. If the obturator foramen is correctly identified in *P*. *wardii*, then it was much larger than that of *Basilosaurus*, *Chrysocetus*, or *Dorudon*. Our interpretation of the innominate of *P*. *wardii* differs from that of Uhen [26] in three ways: (1) we regard Uhen’s ischium as a portion of the ilium; (2) we regard his ilium as the pubic ramus; and (3) we regard his edge of the obturator foramen as a short segment of the outer edge of the innominate. The innominate of *P*. *wardii* is not only different from those of protocetids in lacking a sacral articulation, but it is also different from innominates known for basilosaurines and dorudontines.

Lucas [85], Gidley [89], Kellogg [1], Gol’din [90], Martínez et al. [9], Lambert et al. [86], and Buono et al. [87], interpreted the innominates of *Basilosaurus* and related forms differently from Gingerich et al. [75], who we follow here. The innominate of a basilosaurid differs from the pelvic bone of a modern cetacean in having a well-defined obturator foramen and a recognizable acetabular fossa. The pelvic bone of modern cetaceans lacks these landmarks; is reduced in size; and has the ilium, ischium, and pubis fused or reduced by attrition to a single element.

According to Martínez et al. ([9], p. 130) and other authors, the pelvic bone of modern cetaceans has the orientation typical of quadrupedal mammals (including protocetid archaeocetes), with (1) the ilium anterior to the ischium; and (2) the ischium dorsal to the pubis. On the first point, Martínez et al. cited Struthers [91]—but Struthers did not use the term ilium in reference to cetaceans. Struthers reasoned (page 148), echoing Flower ([92], p. 293), that the modern cetacean pelvic bone is “represented by the ischium alone.” On the second point, Martínez et al. cited Struthers [91] and Andrews [93], but Struthers did not distinguish the pubis from the ischium, and Andrews [93] did not mention either an ischium or a pubis.

The innominates of basilosaurids differ from those of quadrupedal mammals and from those of protocetids in that they no longer articulated with sacral vertebrae, but this does not mean left and right innominates no longer articulated with each other. Surficial bone of the basilosaurid innominate is smooth, except for one rugose surface that resembles the pubic symphysis in a protocetid (compare Fig 13B, 13E and 13F). The basilosaurid symphyseal surface is a little shorter anteroposteriorly than the symphyseal surface in a protocetid of similar size, but this shortening is expected in an element that is itself reduced in size. When left and right innominates are known for the same basilosaurid individual, the left and right symphyseal surfaces match closely in size.

Finally, the natural curvature of basilosaurid innominates (seen in Fig 13D) is similar to curvature in the pubic rami of a protocetid: both follow the curvature of a transverse section of the animal’s ventral body wall where the innominates are located near the base of the tail. This curvature is a functional necessity because left and right acetabula for articulation of the hind limbs, and the midline pubic anchorage for genitalia must all remain near the body surface. The observed curvature would be inexplicable if the innominates were longitudinal elements separated from each other and oriented anteroposteriorly in the lateral wall of the body.

### Locomotion and behavior of pachycetine archaeocetes

Salient features of the FSAC Bouj-200 skeleton and referred specimens of *Antaecetus aithai* relevant to its locomotion and behavior are:

1. the skull is small relative to the size of the vertebrae, and the teeth are relatively small and gracile;
2. vertebrae increase rapidly in size through the thorax, and remain relatively long through the lumbar series;
3. transverse processes on lumbar vertebrae are nearly as long anteroposteriorly as the vertebral centra;
4. most vertebrae and some ribs are conspicuously pachyostotic and osteosclerotic; and
5. rib articulations are cartilaginous and ligamentous, facilitating enlargement and reduction of thorax volume.

*Pachycetus paulsonii* and *P*. *wardii* are similar to *A*. *anthai* in the elongation, pachyostosis, and osteosclerosis of posterior thoracic, lumbar, and anterior caudal vertebrae, but the two *Pachycetus* species appear to have retained larger skulls and more robust teeth.

Skeletal elongation in pachycetines is similar to that in *Basilosaurus* (Fig 11), and the pelvic girdle and hind limb appear to have been similarly modified (Fig 13). We infer from their elongated torso and reduced hind limbs that *Antaecetus* and *Pachycetus* swam, like *Basilosaurus* [84, 94], by undulation of the body as a whole. However, in contrast to *Basilosaurus*, anteroposterior elongation of the transverse processes in *Antaecetus* and *Pachycetus* means that there was little space between adjacent transverse processes for muscle contraction—minimizing the potential for lateral bending and lateral undulation of the vertebral column. With this constraint, swimming in *Antaecetus* and *Pachycetus* was limited to dorsoventral undulation.

*Antaecetus* and *Pachycetus* differ from *Basilosaurus* in having much greater development of bone density: vertebrae as well as ribs are characterized by pachyostosis and osteosclerosis. Ballasting like this may be associated with increased lung volume, and the two together are arguably advantageous in an animal that: (1) feeds in relatively shallow water, at or near the sea bottom; (2) hovers or swims slowly; and (3) relies on free air in the lungs as an oxygen store [95]. Cartilaginous and ligamentous rib articulations may have enabled enlargement of the thorax to increase air intake at the sea surface, and then facilitated collapse of the thorax as air was exhaled to minimize buoyancy on the sea bottom. The locomotor cost of such extensive pachyostosis and osteosclerosis in a swimmer is reduction of the ability to accelerate and maneuver.

From this we infer that pachycetines were probably slow swimmers feeding in the shallow neritic zone of coastal seas. However, *Antaecetus*, with its relatively small and gracile teeth, cannot have fed on plants as sirenians do, nor hard-shelled invertebrates as sea otters do. Small gracile teeth make it unlikely that *Antaecetus* fed on the sea bottom because ingested sediment would make such delicate teeth wear rapidly. As a slow swimmer, *Antaecetus* is not likely to have been a pursuit predator but rather an ambush predator, lying in wait for passing fish or mobile invertebrates of some kind. Mammals require oxygen, so *Antecetus* necessarily rose to the surface from time to time to breathe as modern cetaceans do.

## Conclusions

*Pachycetus paulsonii*, *Pachycetus wardii*, and *Antaecetus aithai* are middle Eocene archaeocetes found in Europe, North America, and Africa, respectively. The three are placed in the new basilosaurid subfamily Pachycetinae. Within Basilosauridae, basilosaurine and pachycetinae genera differ from dorudontines in having posterior thoracic, lumbar, and anterior caudal vertebrae with elongated centra. Pachycetines differ from basilosaurines in having conspicuously pachyostotic vertebrae with thick, dense, laminated cortical bone surrounding a cancellous core. Pachycetinae are also distinctive in having transverse processes on lumbar vertebrae nearly as long anteroposteriorly as the corresponding centrum.

*Antaecetus* is a new genus and the only pachycetine known from a cranium and substantial axial skeleton. It is smaller than *Pachycetus*, and differs in having smaller teeth and possibly a smaller skull relative to its body size. The skull of *A*. *aithai* described here resembles that of *Saghacetus osiris* in size, but lacks the narrowly constricted rostrum of *S*. *osiris*. Teeth of *Antaecetus* are more gracile than those of *Pachycetus* and upper premolars differ in having two rather than three accessory cusps flanking the principal cusp. The vertebral length profile of *Antaecetus* parallels that of *Basilosaurus* in having an inflection between anterior and middle thoracics and another between middle and posterior thoracics. The innominate known for *Pachycetus* is different from that of basilosaurines and dorudontines, but similarly reduced by comparison with earlier protocetids. It is doubtful that *Pachycetus* or any basilosaurid used its reduced hind limbs in locomotion.

We infer that pachycetines were probably sirenian-like, slow, torso- and tail-powered swimmers feeding in the shallow neritic zone of coastal seas, lying in wait to ambush fish and mobile invertebrates, and rising to the surface when necessary to breathe.

## Supporting information

S1 Table Measurements of *Antaecetus aithai* vertebrae in the Faculté des Sciences Ain Chock [FSAC], Casablanca, collection. Specimens come from two localities in southwestern Morocco, Gueran and El Breij. FSAC numbers are given where these have been assigned. Positions represented are *Th*, thoracic; *L*, lumbar; or *Ca*, caudal. Measurements of the centrum are length (L), anterior width (AW), anterior height (AH), posterior width (PW), and posterior height (PH), Measurements of the neural canal are width (NCW), and height (NCH). Measurements of the neural arch are centrum length anterior to the neural arch (NA1), anteroposterior length of the base of the neural arch (NA2), centrum length posterior to the neural arch (NA3), and length of NA2 as a proportion of NA1+NA2+NA3 (NA2P). Measurements of the transverse process are centrum length anterior to the transverse process (TP1), anteroposterior length of the base of the transverse process (TP2), centrum length posterior to the transverse process (TP3), and length of TP2 as a proportion of TP1+TP2+tP3 (TP2P). Numbers in square brackets are interpolated.
(XLSX)
